# Genetic Variants of the Human Thiamine Transporter (*SLC19A3*, THTR2)—Potential Relevance in Metabolic Diseases

**DOI:** 10.3390/ijms26072972

**Published:** 2025-03-25

**Authors:** Edit Szabó, Márton Pálinkás, Balázs Bohár, Botond Literáti-Nagy, László Korányi, Gyula Poór, György Várady, Balázs Sarkadi

**Affiliations:** 1 Institute of Molecular Life Sciences, HUN-REN Research Centre for Natural Sciences, 1117 Budapest, Hungary; szabo.edit@ttk.hu; 2 Department of Rheumatology and Immunology, Semmelweis University, 1023 Budapest, Hungary; mrpalinkas@gmail.com (M.P.); poor.gyula@orfi.hu (G.P.); 3 Department of Internal Medicine and Haematology, Semmelweis University, 1088 Budapest, Hungary; 4 Doctoral School of Biology, Eötvös Loránd University, 1117 Budapest, Hungary; bbazsi41@gmail.com; 5 Drug Research Center, 8230 Balatonfüred, Hungary; botond.literati@gmail.com (B.L.-N.); laszlo.koranyi@drc.hu (L.K.); 6 Doctoral School, Semmelweis University, 1085 Budapest, Hungary

**Keywords:** thiamine, vitamin B1, THTR2, *SLC19A3*, type 2 diabetes, gout, metformin, fedratinib

## Abstract

Thiamine, crucial for energy metabolism, is associated with various human diseases when deficient. We studied how variations in the *SLC19A3* gene, encoding THTR2, a thiamine transporter, may influence type 2 diabetes (T2DM) and gout (arthritis urica, AU). We characterized the *SLC19A3* gene variants using bioinformatics and analyzed DNA samples from controls, T2DM, and gout patients to explore associations with physical/laboratory parameters. In human cells, we used a luciferase reporter assay to assess how these variants affect gene expression. We examined four large haplotypes (H1–4) in this gene, identified lead SNPs for the minor variants (MV), and explored potential transcription factor binding sites. We found that in T2DM patients, H3-MV correlated significantly with impaired glucose metabolism (pHOMA = 0.0189, pHbA1c% = 0.0102), while H4-MV correlated with altered uric acid (*p* = 0.0008) and white blood cell levels (*p* = 0.0272). In AU patients, H3-MV correlated with increased basophil granulocyte levels (*p* = 0.0273). In model cell lines, H3-MV presence increased gene expression (*p* = 0.0351), influencing responses to thiamine depletion and metformin (*p* = 0.0016). Although H4-MV did not directly affect luciferase expression, thiamine and fedratinib co-treatment significantly enhanced gene expression in thiamine-depleted cells (*p* = 0.04854). Our results suggest a connection between selected *SLC19A3* variants and the severity of metabolic diseases or their response to treatment.

## 1. Introduction

Thiamine, or vitamin B1, is an essential co-factor of glucose metabolism in almost all living organisms, and a modulator of neuronal and neuromuscular transmission in vertebrates. In human cells, the active form is thiamine diphosphate, which plays an important role as a co-factor in various metabolic pathways [1,2,3].

The *SLC19A3* gene, encoding thiamine transporter-2 (THTR2), is located in the 36.3 position of the q arm of chromosome 2; the gene has six exons and 13 putative enhancer regions. THTR2 is an SLC (solute carrier)-type thiamine transporter [4,5], and mutations in the *SLC19A3* gene are responsible for extremely rare diseases, biotin-responsive basal ganglia disease and related encephalopathy—see [4]. However, genetic variants and a complex regulation of cellular thiamine transport may play a role in various metabolic disorders. High blood glucose down-regulates THTR2, probably through the Sp1 (specificity protein 1) transcription factor in human renal proximal tubular epithelium [6,7]. Two single nucleotide polymorphisms (SNPs) in the *SLC19A3* gene were also found to be associated with protection against diabetic retinopathy and nephropathy in type 1 diabetes [6,8]. Subsequent in vitro work showed that reduced thiamine availability, in combination with high glucose, impairs thiamine transport primarily via THTR2, both in the retinal and renal cells involved in diabetic microangiopathy [8,9,10]. In the case of THTR2 deficiency, there is no systemic decrease in the thiamine levels, and such a decrease can only be detected in the cerebrospinal fluid [4].

Thiamine uptake by THTR2 was found to be sensitive to changes in pH and the electrochemical gradient, as well as to various drugs [11]. Metformin, widely used as a first-line therapy for type 2 diabetes (T2DM), was shown to be a competing substrate for THTR2-dependent thiamine uptake. Phenformin, chloroquine, verapamil, famotidine and amprolium were also found to inhibit THTR2-mediated uptake of both thiamine and metformin [11]. Thus, THTR2 may play a role in the intestinal absorption and tissue distribution of metformin, as well as for other organic cationic compounds, making this transporter a target for drug–drug and drug–nutrient interactions [5,11,12,13].

The role of THTR2 activity and the potentially related polymorphisms in the *SLC19A3* gene in diabetes have been already suggested [4,8,10], and in our current work we decided to explore in detail the potential genetic alterations modulating THTR2 transporter activity in metabolic diseases. In this work we first focused on T2DM, and identified four major, potentially disease-associated haplotypes in the *SLC19A3* gene. To further explore the metabolic significance of these haplotypes, we analyzed their association with clinical laboratory parameters in T2DM patients. Our findings suggest that *SLC19A3* gene variations may also be implicated in gout (arthritis urica, AU), another metabolic disorder characterized by elevated serum uric acid levels. The deposition of urate crystals in joint tissues induces an inflammatory response, leading to clinical manifestations of gout. T2DM and gout may be connected, as increased insulin concentrations promote uric acid reabsorption through the regulation of ABCG2 (ATP binding cassette G2) or the URAT1 (urate transporter 1) transporters, reducing urinary uric acid extraction and increasing serum uric acid levels [14]. Based on these data, we have also performed an analysis of potential *SLC19A3* gene alterations in patients with the renal overload type of gout [15].

These findings offer new insights into the genetic regulation of THTR2 transporter activity and its potential involvement in metabolic disorders. By identifying clinically relevant *SLC19A3* haplotypes and their effects on gene expression, our study enhances the understanding of the metabolic background of T2DM and gout, shedding light on their underlying pathomechanisms. These results may contribute to advancing disease comprehension and support the development of targeted therapeutic strategies in the future.

## 2. Results

### 2.1. In Silico Haplotype and SNP Analysis in SLC19A3

**Figure 1:**
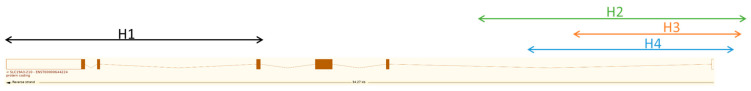
Schematic diagram of the location of the four haplotypes examined. The gene map of *SLC19A3* is from the Ensembl database. H1: Haplotype 1, H2: Haplotype 2, H3: Haplotye 3, H4: Haplotype 4.

By using the 1000 Genomes (1000 G) project [16], we identified and characterized 111 SNPs in *SLC19A3* potentially associated with T2DM (for details see Section 4). These SNPs were analyzed by LDlink, and we identified four large haplotypes (H1, H2, H3, H4) in the related regions (Figure 1). One of these haplotypes (H1) was in exonic regions, and H2–4 were in the predicted promoter regions. Regarding these haplotypes, in each one we identified lead SNPs, then we examined the frequency of the minor variants of these SNPs among control and metabolic disease patients.

Haplotype 1 (H1) includes three exonic regions without a predicted enhancer region. Based on bioinformatic studies, no transcription factor (TF) binding site was found within this haplotype. The minor allele frequency (MAF) value of this minor variant is very low (MAF = 0.01), and due to this low allele frequency, further examination of the haplotype was not performed here (for details see Figure S1 and Table S1).

Haplotype 2 (H2) includes the predicted promoter region and enhancer 5, containing 12 SNPs (Figure S2 and Table S2) The MAF value for the lead SNP of this minor variant is 0.2445, and three SNPs in these regions have a total of 96 potential TF binding sites. The predicted transcription factors are involved in embryonic development (HOX genes) [17] or in the regulation of the nervous system (FoxB1, RAX2) [18,19], but factors affecting the function of the immune system also appear (EBF genes, PROX1, STAT5, RFX1) [20,21,22,23]. There are also binding sites for several transcription factors associated with glucose metabolism, such as NR1H4, RORC, NR2C1 (Table S3) [24,25,26].

In this work, we designated rs6436729 as lead SNP for the minor variant of H2, because most of the potential regulatory factors are predicted to bind to this site (Table S3). The Haplotype variants showed a Hardy–Weinberg equilibrium (HWE, *p* = 0.9784, with Chi-square tests), and the respective MAFs were q(T) = 0.2156, in our sample set (262 samples of studied individuals) (Table S4). Interestingly, the minor allele is mostly T in Europe but A in Africa and Asia.

Haplotype 3 (H3) is the largest haplotype examined in terms of the number of polymorphisms, encompassing 36 SNPs. It is located in the 3rd and 4th enhancer regions of the gene (Figure S3, Tables S5 and S6). In H3, we selected rs34241868 as lead SNP for the minor variant, an intronic variant with a MAF value of 0.1829. The Haplotype variants showed a Hardy–Weinberg equilibrium (*p* = 0.9422, with Chi-square tests), and the respective MAFs were q(A) = 0.0935, in our sample set (Table S4).

In the region of H3, 12 transcription factors are predicted to bind. In the presence of the H3 minor variant, including rs17372552, the Nr5a2 TF cannot bind to this site. This TF plays an important role in bile acid synthesis, cholesterol homeostasis and triglyceride synthesis [27]; thus, the minor variant may cause a potential alteration in these metabolic processes. Another important TF with altered binding to the minor variant in the region of rs34241868, is KLF13 (Kruppel-like transcription factor 13), which has been shown to be a repressive TF in the Sp/KLF family. KLF13 competes with Sp1 for binding to GC boxes and plays a role in B- and T-cell development; thus, the presence of the minor allele may affect the immune system (Table S6) [28,29].

Haplotype 4 (H4) is located in the enhancer regions 1, 7, 8, and 9, and contains 21 SNPs (Figure S4 and Table S7). There are 54 predicted transcription factor binding sites in this region (Table S8) The selected lead SNP for the minor variant is rs55975119, which is an intronic variant. The MAF value for this SNP is 0.102. The Haplotype variants showed a Hardy–Weinberg equilibrium (*p* = 0.6510, with Chi-square tests), and the respective MAFs were q(A) = 0.2080, in our sample set (Table S4).

The predicted TFs binding sites in H4 are mainly involved in hormonal balance (ESR2, NR3C1, NR3C2) [30,31], while GATA-1 [32] and DDIT3 [33] play an important role in erythroid development. The TFs potentially binding to this region, STAT1, Arid5a, and IRF9, play an important role in antiviral immunity (Table S8) [34,35,36].

### 2.2. Experimental SNP Analysis in T2DM Patients—Correlations with Laboratory Data

When analyzing the clinical data in 66 T2DM patients (25 male and 41 female) and 54 age-matched control individuals (29 male and 25 female), as expected, we found that the abdominal circumference, body mass index (BMI), blood glucose, blood insulin, Homeostasis Model Assessment (HOMA) index or hemoglobin A1C (HbA1c), red blood cells (RBC) and white blood cells (WBC) levels were significantly higher in the T2DM patient group, as compared to the control individuals—see Table S9. In order to explore the potential role of the haplotypes in T2DM, we examined the SNPs in the DNA samples of the control and T2DM patients, and analyzed the potential correlation of the minor variants with the key physical and laboratory parameters.

In the case of Haplotype 2, the lead SNP, rs6436729 was analyzed in T2DM patients and controls. We found no significant deviation from the published MAF values in either of these groups (CTRL: 0.1944, T2DM: 0.25). The HWE values were similar in both groups (CTRL: 0.7814, T2DM: 0.7044) (Table S10). The laboratory data showed no significant differences between the individuals carrying the wild-type and the minor variant, respectively, either in the CTRL or in the T2DM group (Table S11).

In the case of Haplotype 3, the MAF value of the examined lead SNP (rs34241868) in the CTRL group was similar to that in the NCBI data (Table S12). The MAF values in the T2DM patient group (0.2045) appeared higher than those in the CTRL group (0.1759), while this difference was not statistically significant. The HWE value appeared to be lower in the T2DM (0.5684) group than in the CTRL group (0.8913), but again, this difference was not statistically significant.

**Figure 2:**
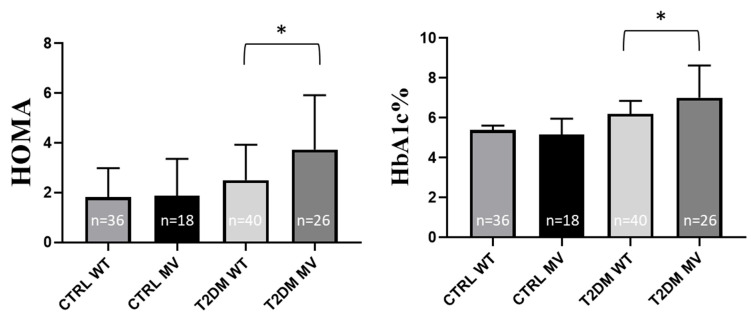
HOMA index, and HbA1c% in the presence of the rs34241868 (Haplotype 3) minor variant (both hetero- and homozygotes), in the groups of CTRL individuals and T2DM patients, respectively. Values are expressed as mean ± SD. The *n* values belonging to each group are shown in the column graphs. The *p* values are calculated by the Mann–Whitney U test. * *p* < 0.05. CTRL: control, T2DM: type 2 diabetes mellitus, WT: wild type, MV: minor variant, HOMA: Homeostasis Model Assessment, HbA1c%: hemoglobin A1c%.

In the following, we analyze the key physical and laboratory parameters with or without the presence of MV of H3 (Table S13). Although blood glucose and blood insulin levels were somewhat higher in the presence of this minor variant in the T2DM group, these differences were not statistically significant (*p* = 0.0756 and *p* = 0.0571, respectively—see Table S13B). However, importantly, the HOMA value (reflecting insulin resistance and β-cell function) was significantly higher in T2DM patients carrying H3-MV (*p* = 0.0189). Also, the HbA1c level, a key indicator for long-term glucose overload, was significantly higher in T2DM patients carrying H3-MV, as compared to patients carrying the wild type allele (*p* = 0.0102). In the control individuals, the presence of H3-MV did not significantly affect this laboratory parameter (Table S14 and Figure 2).

Interestingly, in the control group the abdominal circumference was significantly lower in individuals carrying the minor variant, as compared to those with the wild type alleles (*p* = 0.0186). This parameter in T2DM patients was not different with or without the presence of H3-MV (Table S13B). Other clinical parameters, such as uric acid, inflammatory factors were not significantly different between T2DM patients and the control individuals, regarding the presence or absence of H3-MV (Table S13).

In the case of Haplotype 4, the lead SNP, rs55975119 was analyzed in T2DM patients and controls (Table S15). We found no significant deviation from the published MAF values in either of these groups (CTRL: 0.1018, T2DM: 0.106). The HWE values appeared lower in the T2DM group (0.6601), compared to the control group (0.9198), but this difference was not statistically significant.

**Figure 3:**
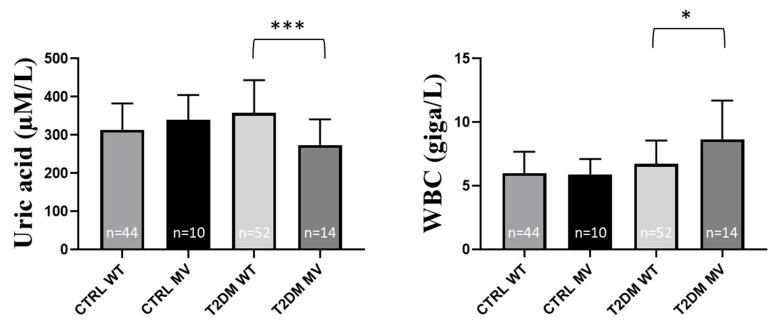
Uric acid levels and white blood cell (WBC) count in the presence of the rs355975119 (haplotype 4) variant (both hetero- and homozygotes), in the groups of CTRL individuals and T2DM patient. Values are expressed as mean ± SD. The *n* values belonging to each group are shown in the column graphs. The *p* values are calculated by the Mann–Whitney U test. * *p* < 0.05, *** *p* < 0.001. CTRL: control, T2DM: type 2 diabetes mellitus, WT: wild type, MV: minor variant, WBC: white blood cells.

The physical parameters were not significantly different in the examined groups (Table S16). Interestingly, in the control individuals the blood glucose level was significantly lower in the presence of H4-MV, (*p* = 0.0474), while this difference was not observed in the T2DM patients (Table S16B). We did not find any differences in the blood insulin, HOMA or HbA1c levels in the studied groups. However, as shown in Table S17 and Figure 3, uric acid levels were significantly lower in the T2DM group of patients carrying H4-MV (*p* = 0.0008). Among the parameters indicating inflammation, the white blood cell count was significantly higher in the T2DM patients carrying H4-MV, as compared to the patients with the wild type allele (*p* = 0.0272). The uric acid levels and the WBC counts were similar in the control individuals with or without the presence of H4-MV (Table S17, Figure 3). Other clinical inflammatory parameters were not significantly different in any of the examined groups (Table S16).

### 2.3. Experimental SNP Analysis in Gout Patients—Correlation with Laboratory Data

We examined the four *SLC19A3* haplotypes in 76 renal overload gout patient (AU) (69 male and 7 female) and 66 age-matched controls (57 male and 9 female) from the same clinic, and analyzed the key labor parameters, such as uric acid (UA), urea, creatinine, red blood cells count (RBC), white blood cells count (WBC), erythrocyte sedimentation rates (ESR), and inflammatory factors. As expected, among these parameters the uric acid levels and the ESR values were significantly higher in the AU group, as compared to the controls. In the case of AU patients, we performed a detailed analysis of the potential correlations of the minor haplotype variants and the laboratory parameters (Table S18).

In the case of Haplotype 2, the lead SNP, rs6436729 was analyzed (Table S19) and we found no significant deviation from the published MAF values in these groups (CTRL: 0.1918, AU: 0.2105). The HWE values were similar in both groups (CTRL: 0.9864, AU: 0.9835).

Interestingly, the laboratory data showed that the ESR values were significantly lower (*p* = 0.0113) in the control patients with H2-MV. No other clinical parameters showed significant correlation with the H2 haplotype, either in the AU patients or the control individuals (Table S20).

In the case of Haplotype 3, the MAF value in the CTRL group was similar to the literature data (Table S21). The MAF value in the AU patient group (0.2632) was somewhat higher than in the control group (0.1986), but this difference was not statistically significant. The HWE values were similar in both groups (CTRL: 0.8908, AU: 0.9939).

**Figure 4:**
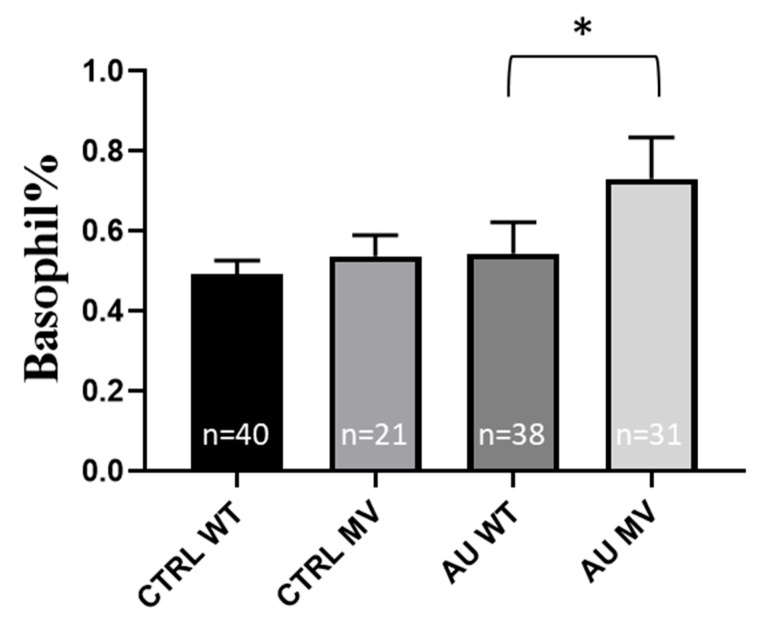
Basophil% in the presence of the rs34241868 (haplotype 3) variant (both hetero- and homozygotes), in the groups of CTRL individuals and gout patient. Values are expressed as mean ± SD. The *n* values belonging to each group are shown in the column graphs. The *p* values are calculated by the Mann–Whitney U test. * *p* < 0.05. CTRL: control, AU: gout, WT: wild type, MV: minor variant.

Among the examined laboratory parameters (Table S22), the basophil granulocyte% value was significantly higher in the AU group with H3-MV (*p* = 0.0273), while there was no such difference in the control group (Table S23 and Figure 4). Other clinical laboratory parameters showed no significant differences in this regard (Table S22).

In the case of Haplotype 4, the lead SNP, rs55975119 was analyzed (Table S24) and the MAF values were lower in both groups (CTRL: 0.089, AU: 0.0724), as compared to the published MAF values (0.1024), but these differences were not statistically significant. The HWE values were apparently lower in the AU group (0.4304), as compared to the control group (0.7278), but again, this difference was not statistically significant.

In the following, we analyze the key physical and laboratory parameters in the presence of H4-MV (Table S25). In the control subjects, in the presence of H4-MV, the mean value of ESR was significantly lower (*p* = 0.0241), while in the AU group carrying this MV a similar tendency was observed, although not reaching statistical significance (*p* = 0.0752). Interestingly, in the relevant control group the lymphocyte% (*p* = 0.0186), eosinophil% (*p* = 0.0308) and creatine levels (*p* = 0.0299) were significantly lower, while the neutrophil counts were higher (*p* = 0.0299) in individuals with H4-MV. In the group of AU patients these differences were not statistically different (Table S25).

### 2.4. Analysis of the Effects of the Selected Haplotypes on Gene Expression in a Luciferase Reporter Assay

In these experiments, we examined the effects of the lead SNP regions of Haplotype 3 (rs34241868) and Haplotype 4 (rs55975119), on gene expression in HEK293 and MCF7 cells, by using a dual-luciferase reporter assay. The effects of thiamine, the antidiabetic drug metformin, and fedratinib (a THTR2 inhibitor) were also studied under normal and thiamine-deficient conditions in the HEK293 cell line.

**Figure 5:**
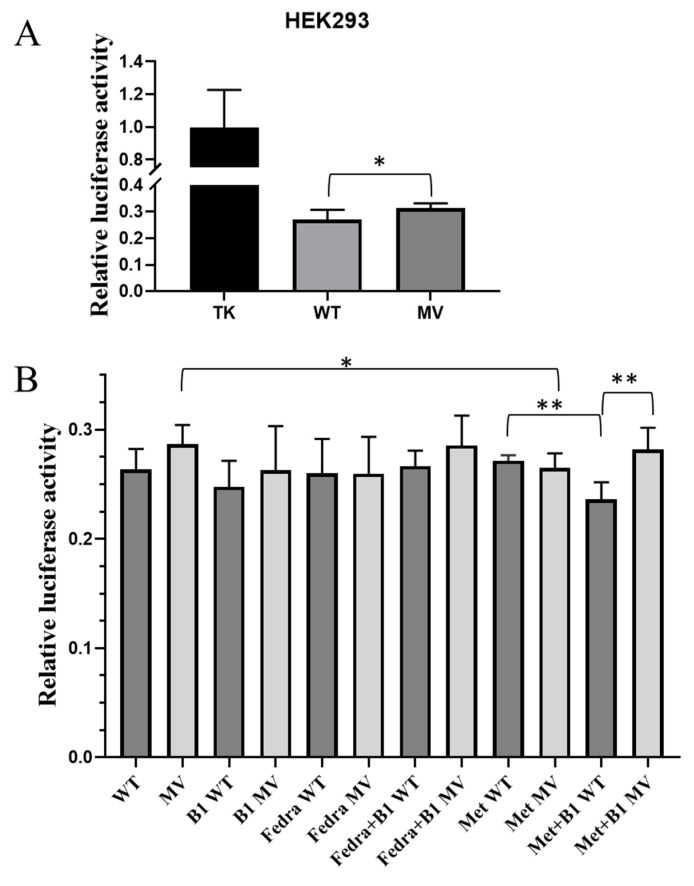
Evaluation of the dual-luciferase assay results. The ratio of the Renilla/normalized Firefly luciferase activity is shown (mean ± SD, *n* = 3, unpaired two-tailed *t* test). (**A**) The effects of Haplotype 3, rs34241868 on luciferase activity. The WT or the rs34241868 versions of the *SLC19A3* H3 regions are expressed in HEK293 cells. (**B**) The role of selected treatments in modulating Renilla luciferase expression in the dual-luciferase assay, performed in thiamine-depleted HEK293 cells. TK: thymidine kinase promoter-containing control vector WT: wild type, MV: minor variant, B1: Vitamin B1, Fedra: fedratinib, Met: metformin * *p* < 0.05, ** *p* < 0.01.

First, we examined the effect of H3-MV (rs34241868 SNP) on luciferase expression. As shown in Figure 5A and Figure S5, when the WT or the minor variant form of the *SLC19A3* cDNA was inserted into the Renilla luciferase vector, we observed very low luciferase expression in both HEK293 and MCF7 cells, as compared to the thymidine kinase (TK) promoter-containing control vector. In the MCF7 cell line, we did not observe any difference in luciferase expression between the WT or the minor variant constructs (Table S26). However, in HEK293 cells, significantly higher expressions from the H3-MV containing construct could be observed, as compared to the wild type construct (*p* = 0.0351—Figure 5A). In HEK293 cells grown in thiamine-depleted media, a similar tendency was observed, while the expression levels were not significantly different between cells containing the WT or the minor variant of the rs34241868 construct (Figure 5B, first 2 columns, and Table S27). Also, in thiamine-depleted HEK293 cells the re-addition of high thiamine (B1) concentrations caused no significant differences in the expression levels driven by any of these constructs (Figure 5B, columns 3 and 4, Table S27). However, under similar conditions, in the case of the H3-MV construct, the addition of thiamine + metformin (last two columns) produced significantly higher luciferase expression (*p* = 0.0016) than that found in the case of the WT variant. Also, in this system, in the case of the H3-MV containing construct, metformin produced significantly lower expression levels (*p* = 0.0018, Figure 5B, column 2 vs. column 10). The addition of fedratinib, a JAK kinase inhibitor, either alone or together with thiamine, had no significant effect in this system (Figure 5B, columns 5–8).

**Figure 6:**
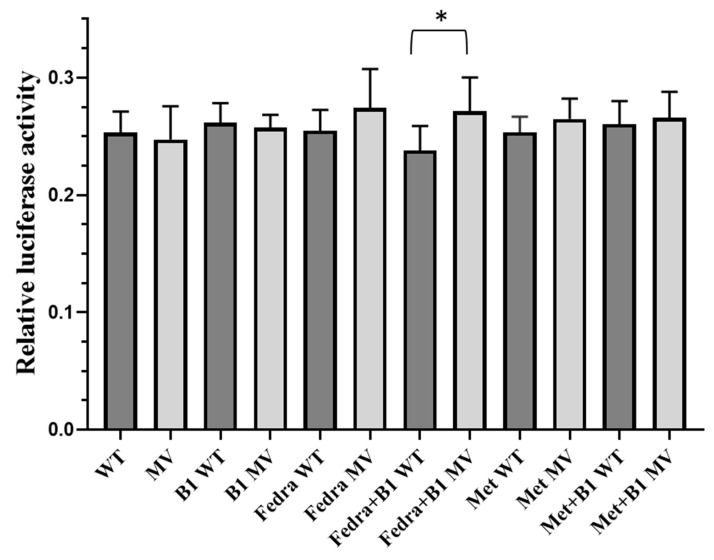
Evaluation of the dual-luciferase assay results. The role of selected treatments in modulating Renilla luciferase expression in the dual-luciferase assay, performed in thiamine-depleted HEK293 cells. The effects of rs55975119 on luciferase activity. The ratio of the Renilla/normalized Firefly luciferase activity is shown (mean ± SD, *n* = 3, unpaired two-tailed *t* test). WT: wild type, MV: minor variant, B1: Vitamin B1, Fedra: fedratinib, Met: metformin. * *p* < 0.05.

In the following experiments, we investigated the effect of the rs55975119 SNP (Haplotype 4) in HEK293 and MCF7 cells, using a dual luciferase reporter assay (Figure S6 and Table S28). Similar to the H3 constructs, in this case we also found low luciferase expression levels compared to the control construct, containing the TK promoter. No differences in the expression levels between the H4 wild-type and H4-MV constructs were found in either of these cell types. The addition of excess thiamine, metformin or fedratinib to the cells did not cause significant changes in the luciferase expression levels. As shown in Figure 6 (and Table S29), in thiamin-depleted HEK293 cells, the addition of excess thiamine or metformin, or these two agents together, produced no alterations in the luciferase expression. However, under these conditions, the addition of fedratinib, together with excess thiamine, caused a significant decrease in luciferase expression only in cells expressing the wild-type form of H4, and not in the case of H4-MV (*p* = 0.04854).

## 3. Discussion

The malabsorption of thiamine (vitamin B1) results in the development of diseases mainly affecting the nervous system. The relatively rare serious conditions can be traced back to the failure of THTR proteins, caused by selected mutations [3,4,5]. The potential role of thiamine transporters in metabolic diseases, including T2DM, is an increasingly investigated area, especially regarding the absorption of vitamin B1 at the cellular level. Currently, diabetes research is mainly focusing on the role of OCT1 vitamin B1 transporter, as metformin treatment, the basic therapy for type 2 diabetes, primarily inhibits the OCT1 transporter [4,5,6,8,10,37,38].

While OCT1 (*SLC22A1*) is one of the key intestinal transporters of thiamine, various retino-, nephro- or neuropathic complications have also been linked to the THTR1 (*SLC19A2*) and the THTR2 (*SLC19A3*) transporters [5,6,11,12,37,38]. The complex role of these transporters in the development and treatments of metabolic diseases is currently mostly unexplored; therefore, in the present work, we have studied the potential relationship between the polymorphic variants of *SLC19A3* and two metabolic diseases, diabetes and gout.

In this work, we first characterized the *SLC19A3* gene variants using in silico bioinformatics methods, and identified four haplotypes (H1–H4) with potential relevance in THTR2 transporter expression. Three of these haplotypes (H2–H4) are located in the promoter region, with numerous potential TF binding sites; thus, these sites may function variably in the genetic variants. Based on our results, the Kruppel-like transcription factor 13 (KLF13) appears to be the most important of the predicted binding sites, located in Haplotype 3 (in the region around rs34241868), and is lost in the H3 minor variant.

KLF13 suppresses gene transcription by competing with the activator Sp1 factor; therefore, in H3-MV, an increased transporter expression may occur (indeed, as shown in the luciferase expression data, such an increased expression was found in HEK cells by H3-MV). SP1 protein expression increases in thiamine-deficient conditions, and Sp1 affects the expression level of *SLC19A3* through a glucose-mediated pathway. This regulation is especially important in insulin-independent cells, including retinal capillary endothelium, pericytes and neuroretina, as these cells cannot regulate glucose uptake and are more vulnerable to hyperglycemic damage [8,28,29]. Thus, complications caused by hyperglycemic damage may occur more often in individuals carrying the H3 minor variant.

Since the minor variant SNPs in *SLC19A3* may affect thiamine transport in complex metabolic conditions, here we studied the effects of these *SLC19A3* gene variants in T2DM and gout patients, as well as their role in related gene expression in human cells by using a luciferase reporter assay.

In exploring the molecular genetic background of T2DM and AU patients, no accumulation of the minor variants of any of the haplotypes was observed. Still, when examining the relationship of laboratory parameters and the presence of these haplotypes, in the case of *SLC19A3*-H3 (lead SNP rs34241868), in T2DM we observed a significant correlation of two important parameters for diabetes monitoring, higher HOMA indices and higher HbA1c levels, in the presence of the minor variant (H3-MV). The HOMA index, derived from fasting glucose and insulin levels, reflects clinical insulin resistance, while HbA1c levels are key indicators for a long-term increase in blood glucose, affecting disease-related complications in T2DM patients.

According to these results, the presence of H3-MV in the *SLC19A3* gene may dispose for a more pronounced diabetic condition. In T2DM patients, the presence of H3-MV also correlated with higher leukocyte numbers, an indicator of a chronic inflammation. Interestingly, in AU patients carrying H3-MV, we found a higher basophil leukocyte count, potentially reflecting a favorable condition, as basophil counts in AU patients are decreasing during the acute phase and normalized during remission [39].

Regarding Haplotype 4 (lead SNP rs55975119), the presence of the minor variant in T2DM patients showed a strong correlation with lower uric acid levels and higher leukocyte counts. In patients suffering from gout (AU), we found no such effect of H4-MV on uric acid levels, while the presence of this haplotype correlated with significantly lower ESR levels, eosinophil% and lymphocyte%.

In analyzing the effects of H2–H4 in T2DM and AU patients, we observed numerous potential effects of the minor variants, while—most probably because of the relatively low number of patients included in this study—these did not reach the level of statistical significance. Similar studies in larger metabolic disease patient groups should help to explore the clinical significance of our initial findings, presented here in the Supplements.

In order to better understand the potential cellular effects of the *SLC19A3* haplo-types, in the following experiments we examined the effects of the H3 and H4 haplotypes in human model cell lines by using a dual-luciferase expression system. In these experiments, we found that in HEK293 cells luciferase expression driven by the H3 minor variant was significantly higher than the expression driven by H3-WT (although this difference was not detectable in the MCF7 cell line). In the case of HEK293 cells grown in thiamine-deficient media, in response to the addition of metformin, the luciferase activity of the cells expressing the H3-MV construct was significantly reduced, while no such difference was observed in HEK293 cells carrying the H3-WT construct. In addition, when metformin and vitamin B1 were added together to thiamine-depleted HEK293 cells, luciferase expression driven by the H3 wild-type construct was significantly decreased, while there was no such decrease in the case of the construct carrying H3-MV. These data indicate a significantly different response to thiamine and metformin in the presence of these H3 variants.

In the case of Haplotype 4 (rs55975119), in thiamine-depleted HEK cells containing the H4-WT construct, luciferase expression was significantly lower in response to the combination of the JAK inhibitor fedratinib and thiamine, as compared to the expression driven by the H4-MV construct. Fedratinib is in clinical use e.g., in Wernicke’s encephalopathy and in diabetic patients developing myelofibrosis. It has been shown that fedratinib, by inhibiting the *SLC19A3* transporter, prevents the uptake of vitamin B1; thus, thiamine levels have to be normalized before using fedratinib [40,41]. Our results shown here indicate that the presence of the genetic variants in H4 of *SLC19A3* may modulate this clinical response.

Since the haplotypes studied in this work are located within intronic regions, protein expression and phenotypic effects are likely to manifest in a tissue-specific manner by epigenetic regulation through variable transcription factors. While proper animal models are currently unavailable for studying this complex regulation and disease connections, human studies in patients with diabetes indicate their potential importance. Porta et al. [8] and Larkin et al. [42] have studied a connection between glucose regulation and *SLC19A3* in diabetes, and a potential connection with transcription factors, e.g., Sp1 was noted. Regarding the rs34241868 polymorphism, a potential binding site for the transcription factor KLF13 is present, which competes with the Sp1 transcription factor for binding to GC-boxes. These data suggest that Haplotype 3 may be involved in in vivo glucose metabolism.

A limitation of our study is that we did not include blood thiamine level or erythrocyte transketolase activity measurements. However, based on literature data, the reduction linked to the THTR2 transporter could not be detected in blood samples, only in the cerebrospinal fluid [4]. An additional limitation of this study is the relatively low sample size of each group. We plan to confirm the significant differences observed here by examining a larger group of patients, especially with disease complications (e.g., retinopathy, neuropathy). In spite of these limitations, our work may draw attention to the potential benefits of the administration of complex B vitamins alongside metformin therapy, particularly for individuals harboring these polymorphisms.

## 4. Materials and Methods

### 4.1. Bioinformatic Analysis of Candidate SLC19A3 SNPs in T2DM

From the T2D Knowledge Portal (https://t2d.hugeamp.org/ (accessed on 23 June 2020)), a curated T2DM-associated SNP set was retrieved and filtered by the 1000 G project (https://www.ncbi.nlm.nih.gov/ (accessed on 23 June 2020)) [16]. SNPs in high linkage disequilibrium (LD) with T2DM SNPs were collected using the web-based application LDlink (https://ldlink.nih.gov/?tab=home (accessed on 23 June 2020)) and filtered by European sub-population (CEU, TSI, FIN, GBR, IBS) LD criteria (r^2^ > 0.8 and D’ = 1). The promoter and enhancer regions of the *SLC19A3* gene were identified by the Ensembl genome browser (https://www.ensembl.org/index.html (accessed on 23 June 2020)). MAF was determined from the NCBI database (https://www.ncbi.nlm.nih.gov/ (accessed on 23 June 2020)).

For the investigation of the transcription factor binding sites (TFBS) we used the matrix scan function of the Regulatory Sequence Analysis Tools (RSAT, https://rsat.france-bioinformatique.fr/teaching/RSAT_portal.html (accessed on 23 June 2020)). In using this tool, we filtered the 100-nucleotide-long regions around the selected SNPs for both the reference and mutant sequences. We have also downloaded the recent version of the position weight matrices (PWM) for the transcription factors from the Jaspar database (http://jaspar.genereg.net (accessed on 23 June 2020)) in order to identify the predicted TF binding sites. The matrix scan module calculated the *p* value for TFBS (*p* < 0.0005), and we analyzed the unique TFBS that were present only in the reference or alternative sequences. These transcription factors were characterized based on The Human Protein Atlas (https://www.proteinatlas.org/, version 23.0) and literature data.

The lead SNP within a given haplotype was identified based on the number of predicted transcription factors capable of binding to the respective genomic region. Additionally, the classification of the region (e.g., predicted enhancer or promoter) was considered, as determined by Ensembl annotations.

### 4.2. Analysis of Genetic Samples from Patients in the Study

Clinical and laboratory data and blood samples of T2DM patients were obtained from the Drug Research Centre (DRC, Balatonfüred, Hungary). The diagnosis of T2DM was established according to the criteria of the American Diabetes Association (ADA). Based on these criteria, individuals with an HbA1c% exceeding 6% were included in the patient group. The age-matched healthy volunteers were selected from visitors at the clinic not suffering from diabetes or related metabolic diseases. The patients received metformin-based medication [43,44].

The blood samples from AU patients were collected at the National Institute of Locomotor Diseases and Disabilities, (from 1 March 2025 Department of Rheumatology and Immunology, Semmelweis University, Budapest, Hungary). Clinical classification of gout was based on the EULAR criteria. Controls (CTRL) were age-matched healthy volunteers, not suffering of hyperuricemia or gout. The patients received allopurinol-based medication [15].

The study was conducted in accordance with the Declaration of Helsinki and with the permission of the Scientific and Research Committee of the Medical Research Council, Hungary (ETT TUKEB references: 2367-1/2019/EKU and 41006-1/2013/EKU). All methods were performed in accordance with the relevant guidelines and regulations. All control subjects and patients in the study gave informed consent to participate in this research and their privacy and identities were fully protected in the manuscript.

### 4.3. Genetic Analysis and Vector Construction

Genomic DNA was purified from 300 μL of EDTA-anticoagulated blood samples with Puregene Blood Kit (Qiagen, Hilden, Germany). TaqMan-based qPCR reactions for *SLC19A3* SNPs (details see in Table 1) were performed in a StepOnePlus device (Applied Biosystems, Waltham, MA, USA) with premade assay mixes and a master mix (cat. 4371353) from ThermoFisher. TaqMan probe specificity was verified by sequencing.

The dual luciferase assay was only tested in the Haplotype 3 (rs34241868) and Haplotype 4 (rs55975119) regions. For this experiment, different regions of the *SLC19A3* gene (Figure S7) were cloned into pMCS-Green-Renilla plasmid (ThermoFisher, Waltham, MA, USA cat: 16152) without additional upstream promoter sequences, by using standard restriction enzyme-based cloning. Sequences were amplified from DNA samples extracted from patients’ DNA carrying the WT or the SNPs in homozygous forms. We used the following primers:
1868 For5′-CGT CAG AAT TCT TTC CTT CAG TCA TTA TTG C-3′1868 Rev5′-CGT CGG ATC CCT GAT CTA CGA ATG TAC CC-3119 For5′-AGC TGA ATT CCA TCT ATC CAG TGA CAA CC-3′119 Rev5′-CGC TGG ATC CTG AGA CCT AGG ACT ATG C-3′

### 4.4. Cell Lines and Dual Luciferase Assay

HEK293 cells (from a human embryonic kidney kindly gifted from Tamás I. Orbán, HUN-REN TTK) were grown in DMEM medium with high glucose and GlutaMax (Gibco, ThermoFisher, Waltham, MA, USA, cat. 31966021) completed with 10% FBS (Gibco, ThermoFisher, Waltham, MA, USA, cat. 10500064) and 0.1% gentamycin (Gibco, ThermoFisher, Waltham, MA, USA, cat. 15710080) at 37 °C (5% CO_2_). Thiamine-depleted HEK293 cells were grown in M199 medium (Gibco, ThermoFisher, Waltham, MA, USA, cat. 41150020) completed with 10% FBS and 0.1% gentamycin at 37 °C (5% CO_2_). This latter medium (thiamine concentration: 0.03 µM) contains about 400 times less thiamine-hydrochloride compared to the basic medium (thiamine concentration: 11.9 µM). Since FBS may also contain a small but variable amount of vitamin B1, the same amount of FBS was added from the same lot number of the product to each media. MCF7 cells (a human breast cancer cell line gifted from Gergő Szakács, HUN-REN TTK) were grown in RPMI medium with high glucose and GlutaMax (Gibco, Waltham, MA, USA, cat. 61870010) completed with 10% FBS and 0.1% gentamycin at 37 °C (5% CO_2_). Transient transfection of HEK293 and MCF7 cells was carried out with Turbofect (ThermoFisher, Waltham, MA, USA, cat. R0531), according to the manufacturers’ protocol.

Drug treatments were carried out 24 h after transfection in completed DMEM medium. The cells were subjected to 10 µM thiamine-hydrochloride (Sigma-Aldrich-Merck, St. Louis, MO, USA, cat T4625), 5µM fedratinib (Selleckchem, Houston, TX, USA, cat. S2736) or 1,1-Dimethylbiguanide hydrochloride (metformin, Sigma-Aldrich-Merck, St. Louis, MO, USA, cat. D150959) for 24 h prior to the experiments.

The dual-luciferase reporter assay system (Pierce, ThermoFisher, Waltham, MA, USA, cat. 16185) was used according to the manufacturer’s guidelines for 96-well cultured cells. Reagents were added manually in 96-well white plates to the cell lysates, and incubated for 3 min at room temperature in the dark before reading the signals in a VictorX3 Multilabel Plate Reader (Perkin-Elmer, Waltham, MA, USA). At least four technical and three biological parallels were performed in all cases. The data were normalized to pCMV-Red-Firefly signals (vector from ThermoFisher, Waltham, MA, USA, cat. 16156).

### 4.5. Statistical Analysis

The clinical and laboratory data are expressed as mean ± standard error (SD). Statistical analysis was conducted by GraphPad Prism (version 8.0.1, GraphPad Software, Boston, MA, USA). Normal distribution of the variables was checked by an Anderson–Darling test. The physical parameters, such as height, weight, BMI, abdominal circumference, showed a normal distribution, and in these cases the Student *t* test (2-group analysis) or ANOVA (multigroup analysis) statistical tests were used. The investigated laboratory parameters did not show a normal distribution, so in these cases the Mann–Whitney U test (2 groups) or Kruskal–Wallis (in the case of multigroups) analysis was used. Two component analyses (Student *t* test or Mann–Whitney U test) were used if the *p* values of multigroup analyses (ANOVA or Kruskal–Wallis tests) were below 0.1. The level of significance was set at *p* < 0.05. The number of patients (*n*) involved in each analysis is indicated in the respective tables.

## 5. Conclusions

Our study provides novel insights into the genetic and functional relevance of *SLC19A3* variants in metabolic diseases, particularly type 2 diabetes and gout. We were the first to identify the four major haplotypes in this gene, determine the lead SNPs of the minor variants, and explore potential transcription factor binding sites. Through genetic association studies, and functional assays, we identified significant correlations between specific *SLC19A3* haplotypes and key metabolic parameters. We identified haplotypes H3-MV and H4-MV as potential modulators of glucose metabolism, uric acid levels, and immune cell profiles, suggesting a role for *SLC19A3* in studied diseases pathophysiology. The increased gene expression observed in H3-MV carrier cells under thiamine depletion and metformin treatment further supports the functional relevance of these variants in cellular responses to metabolic stress. Similarly, the H4 minor variant showed sensitivity to fedratinib treatment in thiamine-depleted conditions, further highlighting the clinical relevance of these polymorphisms. While our findings underscore the potential role of *SLC19A3* in metabolic regulation, larger patient cohorts and additional in vitro studies are needed to fully elucidate the impact of these variants on studied diseases development and treatment responses. Our results suggest that personalized approaches, including targeted vitamin B1 supplementation, may be beneficial for individuals carrying these genetic variants, particularly in the context of metformin therapy for diabetes and potential immune modulation in gout.

## Figures and Tables

**Table 1:** SNPs and DNA sequences of the probes used in qPCR experiments.

SNP	Assay ID	Context Sequence [VIC/FAM]
rs6436729	C__26173496_10	GCATGGTGGTATATGCCTGTAGTCC[T/A]AGCTACTGAGAAGCTGAGGCAGGAA
rs34241868	C__32730488_10	AGAATATACCCCACACCTGAACAGA[C/A]CCATTCACAAGATAATGTAGCTTAT
rs55975119	C__88465680_10	TCTCACTTCCTAGCTGCATGCCTTA[A/C]AGGACAGAAAGGTCCTCAGATCTCC

## Data Availability

The datasets generated and analyzed during the current study are available from the corresponding authors on reasonable request.

## Supplementary Materials

The following supporting information can be downloaded at: https://www.mdpi.com/article/10.3390/ijms26072972/s1. Ref. [45] is cited in Supplementary Materials File.

## Abbreviations

1000 G	1000 Genomes
ABCG2	ATP binding cassette G2
ADA	American Diabetes Association
Arid5a	AT-Rich Interaction Domain 5A
AU	arthritis urica, gout
BMI	body mass index
CTRL	control
DDIT3	DNA damage-inducible transcript 3
DNA	deoxyribonucleic acid
EBF genes	early B-cell factors
ESR	erythrocyte sedimentation rates
ESR2	Estrogen Receptor 2
Fedra	fedratinib
FoxB1	Forkhead Box B1
GATA-1	GATA-BINDING PROTEIN 1
HbA1c	hemoglobin A1C
HOMA	Homeostasis Model Assessment
HOX genes	subset of homeobox genes
HWE	Hardy–Weinberg equilibrium
IRF9	Interferon regulatory factor 9
JAK	Janus kinase
KLF13	Kruppel-like transcription factor 13
LD	linkage disequilibrium
MAF	minor allele frequency
met	metformin
MV	minor variant
NR1H4	nuclear receptor subfamily 1 group H member 4
NR2C1	nuclear receptor subfamily 2, group C, member 1
NR3C1	nuclear receptor subfamily 3, group C, member 1
NR3C2	nuclear receptor subfamily 3, group C, member 2
PROX1	Prospero homeobox protein 1
PWM	position weight matrices
RAX2	Retina And Anterior Neural Fold Homeobox 2
RBC	red blood cells
RFX1	Regulatory Factor X1
RORC	RAR-related orphan receptor gamma
SD	standard deviation
*SLC19A3*	*solute carrier 19A3*
SNP	single nucleotide polymorphisms
SP1	specificity protein 1*
STAT1	Signal transducer and activator of transcription 1
STAT5	Signal transducer and activator of transcription 5
T2DM	type 2 diabetes
TF	transcription factor
TFBS	transcription factor binding sites
THTR2	thiamine transporter-2
UA	uric acid
URAT1	urate transporter 1
WBC	white blood cells
WT	wild type
