# Supplementary material for: Genetic Variants of the Human Thiamine Transporter (SLC19A3, THTR2)—Potential Relevance in Metabolic Diseases
Source: Int J Mol Sci. 2025 Mar 25;26(7):2972. doi: 10.3390/ijms26072972 (PMC11988879; doi:10.3390/ijms26072972)
Supplement: Supplementary file 1 [file ijms-26-02972-s001.zip › ijms-3468465-supplementary.pdf]

Supplementary information

for

Genetic variants of the human thiamine transporter (*SLC19A3*, THTR2) - potential relevance in metabolic diseases

by

Edit Szabó, Márton Pálinkás, Balázs Bohár, Botond Literáti-Nagy, László Korányi, Gyula Poór, György Várady and Balázs Sarkadi

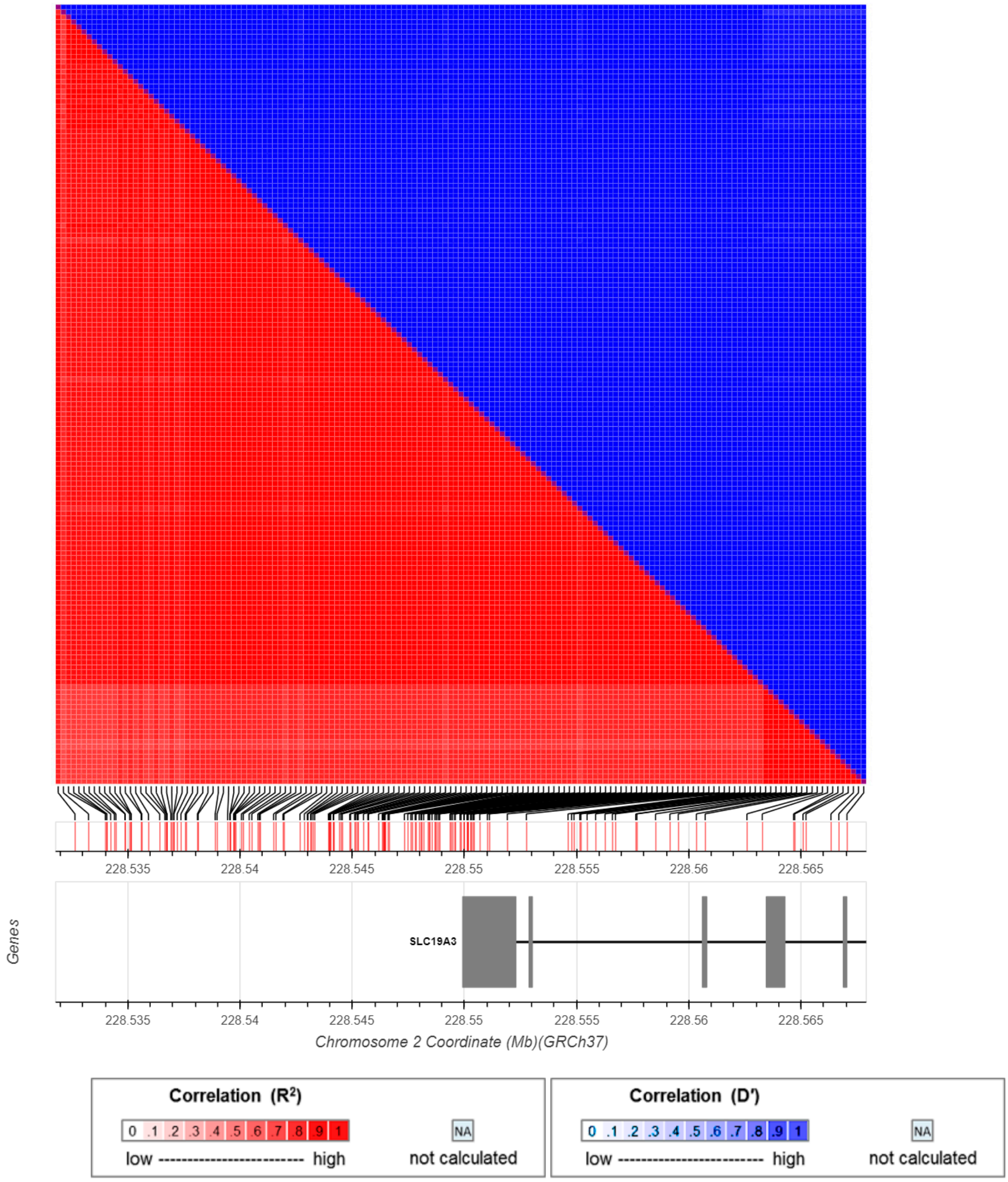

Figure S1. Haplotype 1 Interactive heatmap matrix of Linkage Disequilibrium Calculations: D′ values are represented by the blue squares and R^2 values are represented by the red squares.

Table S1. Characterization of SNPs belonging to the haplotype 1.

RS ID	Position (GRCh37)	Alleles	MAF	Distance	D'	R^2	Correlated Alleles
**rs10498220**	228536712	(G/A)	0,0109	9014	1	0,9082	C=G,T=A
**rs10544518**	228546466	(TC/-)	0,0099	740	1	1	C=TC,T=-
**rs111236896**	228549803	(G/C)	0,0099	4077	1	1	C=G,T=C
**rs111409314**	228550117	(A/G)	0,0099	4391	1	1	C=A,T=G
**rs111436532**	228534234	(G/A)	0,0109	11492	1	0,9082	C=G,T=A
**rs111445989**	228555141	(C/T)	0,0099	9415	1	1	C=C,T=T
**rs111467219**	228551113	(T/C)	0,0099	5387	1	1	C=T,T=C
**rs111510401**	228559528	(T/C)	0,0119	13802	1	0,8317	C=T,T=C
**rs111572681**	228556265	(C/T)	0,0119	10539	1	0,8317	C=C,T=T
**rs111618899**	228564663	(C/G)	0,0119	18937	1	0,8317	C=C,T=G
**rs111765773**	228539807	(C/T)	0,0099	5919	1	1	C=C,T=T
**rs111799560**	228544014	(C/A)	0,0099	1712	1	1	C=C,T=A
**rs111856941**	228565218	(T/C)	0,0119	19492	1	0,8317	C=T,T=C
**rs112030681**	228544499	(C/T)	0,0099	1227	1	1	C=C,T=T
**rs112114926**	228555189	(C/T)	0,0099	9463	1	1	C=C,T=T
**rs112219909**	228566330	(T/C)	0,0119	20604	1	0,8317	C=T,T=C
**rs112322717**	228548555	(T/C)	0,0099	2829	1	1	C=T,T=C
**rs112333340**	228547324	(C/T)	0,0099	1598	1	1	C=C,T=T
**rs112334929**	228549473	(C/T)	0,0099	3747	1	1	C=C,T=T
**rs112560852**	228564718	(T/C)	0,0119	18992	1	0,8317	C=T,T=C
**rs112697749**	228549592	(G/A)	0,0099	3866	1	1	C=G,T=A
**rs112974691**	228544039	(T/G)	0,0099	1687	1	1	C=T,T=G
**rs113016446**	228544558	(A/G)	0,0099	1168	1	1	C=A,T=G
**rs113201675**	228541495	(C/-)	0,0099	4231	1	1	C=C,T=-
**rs113289674**	228547474	(C/T)	0,0099	1748	1	1	C=C,T=T
**rs113294850**	228545697	(C/T)	0,0099	29	1	1	C=C,T=T
**rs113330738**	228540856	(C/T)	0,0099	4870	1	1	C=C,T=T
**rs113398205**	228554774	(C/T)	0,0099	9048	1	1	C=C,T=T
**rs1134281**	228550150	(G/A)	0,0099	4424	1	1	C=G,T=A
**rs1134282**	228550139	(C/A)	0,0099	4413	1	1	C=C,T=A
**rs113452011**	228549407	(G/A)	0,0099	3681	1	1	C=G,T=A
**rs113499955**	228549567	(T/C)	0,0099	3841	1	1	C=T,T=C
**rs113610712**	228556727	(-/A)	0,0119	11001	1	0,8317	C=-,T=A
**rs113664962**	228548762	(A/G)	0,0099	3036	1	1	C=A,T=G
**rs113876064**	228555846	(C/T)	0,0119	10120	1	0,8317	C=C,T=T
**rs113896991**	228548687	(-/T)	0,0099	2961	1	1	C=-,T=T
**rs113939016**	228540793	(T/C)	0,0099	4933	1	1	C=T,T=C
**rs113979210**	228550160	(C/T)	0,0099	4434	1	1	C=C,T=T
**rs114055204**	228549983	(A/G)	0,0099	4257	1	1	C=A,T=G
**rs114520350**	228554615	(G/A)	0,0099	8889	1	1	C=G,T=A
**rs115041609**	228555467	(C/T)	0,0099	9741	1	1	C=C,T=T
**rs115060000**	228556590	(A/C)	0,0119	10864	1	0,8317	C=A,T=C
**rs115320284**	228549981	(A/G)	0,0099	4255	1	1	C=A,T=G
**rs115705488**	228532643	(G/A)	0,0099	13083	1	1	C=G,T=A
**rs12105323**	228550373	(C/G)	0,0099	4647	1	1	C=C,T=G
**rs12105610**	228550686	(A/G)	0,0099	4960	1	1	C=A,T=G
**rs12105620**	228548897	(T/C)	0,0099	3171	1	1	C=T,T=C
**rs12105737**	228550429	(T/C)	0,0099	4703	1	1	C=T,T=C
**rs139091057**	228565087	(A/-)	0,0119	19361	1	0,8317	C=A,T=-
**rs139659526**	228543983	(T/C)	0,0099	1743	1	1	C=T,T=C
**rs1400039**	228536915	(G/A)	0,0109	8811	1	0,9082	C=A,T=G
**rs142996600**	228537199	(AAAAG/-)	0,0099	8527	1	1	C=AAAAG,T=-
**rs147381371**	228543976	(T/C)	0,0099	1750	1	1	C=T,T=C
**rs148387949**	228543173	(C/T)	0,0099	2553	1	1	C=C,T=T
**rs16823825**	228536649	(A/G)	0,0109	9077	1	0,9082	C=A,T=G
**rs187651830**	228548689	(A/T)	0,0099	2963	1	1	C=A,T=T
**rs192332940**	228534882	(C/T)	0,0109	10844	1	0,9082	C=C,T=T
**rs200115608**	228543250	(C/-)	0,0099	2476	1	1	C=C,T=-
**rs200346034**	228543143	(C/T)	0,0099	2583	1	1	C=C,T=T
**rs2203528**	228534061	(C/A)	0,0109	11665	1	0,9082	C=A,T=C
**rs2203529**	228534059	(C/A)	0,0109	11667	1	0,9082	C=A,T=C
**rs2396466**	228540525	(A/G)	0,0109	5201	1	0,9082	C=G,T=A
**rs28395237**	228548841	(C/T)	0,0099	3115	1	1	C=C,T=T
**rs34507036**	228560728	(A/G)	0,0119	15002	1	0,8317	C=A,T=G
**rs375632524**	228539566	(TTTG/-)	0,0099	6160	1	1	C=TTTG,T=-
**rs4513280**	228536741	(G/A)	0,0109	8985	1	0,9082	C=A,T=G
**rs56663417**	228539764	(C/T)	0,0099	5962	1	1	C=C,T=T
**rs57190409**	228539554	(A/G)	0,0099	6172	1	1	C=A,T=G
**rs57582587**	228541915	(A/-)	0,0099	3811	1	1	C=A,T=-
**rs57696054**	228544923	(T/C)	0,0099	803	1	1	C=T,T=C
**rs57775446**	228539558	(G/A)	0,0099	6168	1	1	C=G,T=A
**rs57886606**	228541596	(GTGTGTGT/-)	0,0099	4130	1	1	C=GTGTGTGT,T=-
**rs57918741**	228545155	(A/G)	0,0099	571	1	1	C=A,T=G
**rs58166157**	228542999	(T/G)	0,0099	2727	1	1	C=T,T=G
**rs58386964**	228539725	(C/T)	0,0099	6001	1	1	C=C,T=T
**rs58533431**	228544888	(T/C)	0,0099	838	1	1	C=T,T=C
**rs58871182**	228539714	(A/G)	0,0099	6012	1	1	C=A,T=G
**rs58934450**	228545264	(A/G)	0,0099	462	1	1	C=A,T=G
**rs58957721**	228542864	(T/G)	0,0099	2862	1	1	C=T,T=G
**rs59143032**	228543327	(T/A)	0,0099	2399	1	1	C=T,T=A
**rs60224058**	228545231	(T/C)	0,0109	495	1	0,9082	C=T,T=C
**rs60263223**	228538102	(C/G)	0,0099	7624	1	1	C=C,T=G
**rs60318365**	228545108	(C/T)	0,0099	618	1	1	C=C,T=T
**rs60790986**	228538133	(C/G)	0,0099	7593	1	1	C=C,T=G
**rs61280925**	228543938	(T/G)	0,0099	1788	1	1	C=T,T=G
**rs62189847**	228533248	(C/T)	0,0119	12478	1	0,8317	C=T,T=C

RS ID	Position (GRCh37)	Alleles	MAF	Distance	D'	R^2	Correlated Alleles
**rs6721696**	228560332	(T/C)	0,0119	14606	1	0,8317	C=T,T=C
**rs6728344**	228567046	(T/C)	0,0119	21320	1	0,8317	C=T,T=C
**rs73084836**	228533997	(C/T)	0,0109	11729	1	0,9082	C=C,T=T
**rs73084840**	228534401	(A/G)	0,0109	11325	1	0,9082	C=A,T=G
**rs73084841**	228534469	(T/A)	0,0109	11257	1	0,9082	C=T,T=A
**rs73084847**	228535082	(A/G)	0,0109	10644	1	0,9082	C=A,T=G
**rs73088831**	228536971	(C/T)	0,0099	8755	1	1	C=C,T=T
**rs73088833**	228537051	(A/G)	0,0109	8675	1	0,9082	C=A,T=G
**rs73088834**	228537365	(T/C)	0,0099	8361	1	1	C=T,T=C
**rs73088838**	228537561	(C/T)	0,0099	8165	1	1	C=C,T=T
**rs73088840**	228537605	(A/C)	0,0099	8121	1	1	C=A,T=C
**rs73088852**	228540060	(G/T)	0,0099	5666	1	1	C=G,T=T
**rs73088853**	228540144	(G/A)	0,0099	5582	1	1	C=G,T=A
**rs73088859**	228540406	(C/T)	0,0099	5320	1	1	C=C,T=T
**rs73088861**	228540900	(G/A)	0,0109	4826	1	0,9082	C=G,T=A
**rs73088864**	228541961	(C/T)	0,0099	3765	1	1	C=C,T=T
**rs73088865**	228542672	(T/C)	0,0099	3054	1	1	C=T,T=C
**rs73088869**	228543026	(C/T)	0,0099	2700	1	1	C=C,T=T
**rs73088879**	228544156	(T/C)	0,0099	1570	1	1	C=T,T=C
**rs73088880**	228544420	(T/C)	0,0099	1306	1	1	C=T,T=C
**rs73088885**	228545499	(G/C)	0,0099	227	1	1	C=G,T=C
**rs74372767**	228550274	(C/T)	0,0099	4548	1	1	C=C,T=T
**rs74387373**	228566682	(T/G)	0,0119	20956	1	0,8317	C=T,T=G
**rs74621178**	228536418	(G/A)	0,0099	9308	1	1	C=G,T=A
**rs74714426**	228549848	(C/G)	0,0099	4122	1	1	C=C,T=G
**rs74800339**	228557686	(C/T)	0,0119	11960	1	0,8317	C=C,T=T
**rs74971746**	228552761	(G/T)	0,0099	7035	1	1	C=G,T=T
**rs75032617**	228534873	(A/G)	0,0109	10853	1	0,9082	C=A,T=G
**rs75540593**	228548423	(A/G)	0,0109	2697	1	0,9082	C=A,T=G
**rs7564184**	228539449	(A/G)	0,0099	6277	1	1	C=A,T=G
**rs7576260**	228535623	(G/A)	0,0099	10103	1	1	C=G,T=A
**rs7578736**	228546372	(C/A)	0,0099	646	1	1	C=C,T=A
**rs7578840**	228546339	(G/A)	0,0099	613	1	1	C=G,T=A
**rs7578945**	228546434	(G/T)	0,0099	708	1	1	C=G,T=T
**rs7579047**	228535921	(C/A)	0,0109	9805	1	0,9082	C=C,T=A
**rs7579160**	228546593	(G/A)	0,0099	867	1	1	C=G,T=A
**rs75798521**	228558518	(A/G)	0,0119	12792	1	0,8317	C=A,T=G
**rs75820505**	228535144	(T/C)	0,0099	10582	1	1	C=T,T=C
**rs7590225**	228538893	(C/G)	0,0099	6833	1	1	C=C,T=G
**rs7590334**	228538976	(C/T)	0,0099	6750	1	1	C=C,T=T
**rs7592758**	228546399	(T/C)	0,0099	673	1	1	C=T,T=C
**rs75968368**	228537043	(C/A)	0,0109	8683	1	0,9082	C=C,T=A
**rs75995475**	228544172	(C/T)	0,0099	1554	1	1	C=C,T=T
**rs7604813**	228546175	(A/T)	0,0099	449	1	1	C=A,T=T
**rs7605079**	228546456	(A/G)	0,0099	730	1	1	C=A,T=G
**rs7605291**	228546649	(A/G)	0,0099	923	1	1	C=A,T=G
**rs76100632**	228550300	(T/C)	0,0099	4574	1	1	C=T,T=C
**rs76750729**	228548063	(T/A)	0,0099	2337	1	1	C=T,T=A
**rs76872123**	228546443	(G/T)	0,0099	717	1	1	C=G,T=T
**rs77535228**	228548375	(C/A)	0,0099	2649	1	1	C=C,T=A
**rs77564432**	228547984	(A/T)	0,0099	2258	1	1	C=A,T=T
**rs77815504**	228544173	(A/G)	0,0099	1553	1	1	C=A,T=G
**rs78550805**	228559154	(C/T)	0,0119	13428	1	0,8317	C=C,T=T
**rs78560894**	228551023	(C/T)	0,0099	5297	1	1	C=C,T=T
**rs78803281**	228549356	(T/C)	0,0099	3630	1	1	C=T,T=C
**rs78818006**	228547675	(A/C)	0,0099	1949	1	1	C=A,T=C
**rs78932818**	228548184	(A/C)	0,0099	2458	1	1	C=A,T=C
**rs79144487**	228554881	(A/G)	0,0099	9155	1	1	C=A,T=G
**rs79387463**	228548093	(A/T)	0,0099	2367	1	1	C=A,T=T
**rs79627154**	228536740	(C/T)	0,0099	8986	1	1	C=C,T=T
**rs79720535**	228548454	(C/T)	0,0099	2728	1	1	C=C,T=T
**rs79738780**	228547612	(T/C)	0,0099	1886	1	1	C=T,T=C
**rs79823446**	228547841	(C/G)	0,0099	2115	1	1	C=C,T=G
**rs80055005**	228557635	(G/A)	0,0119	11909	1	0,8317	C=G,T=A
**rs80120369**	228547806	(A/G)	0,0099	2080	1	1	C=A,T=G
**rs80307329**	228551910	(G/A)	0,0099	6184	1	1	C=G,T=A
**rs970477**	228535594	(G/A)	0,0109	10132	1	0,9082	C=A,T=G
**rs970478**	228535142	(C/T)	0,0109	10584	1	0,9082	C=T,T=C
**rs112266944**	228563270	(T/C)	0,0129	682	1	1	T=T,C=C
**rs113876064**	228555846	(C/T)	0,0119	6742	1	0,9221	T=C,C=T
**rs111572681**	228556265	(C/T)	0,0119	6323	1	0,9221	T=C,C=T
**rs115060000**	228556590	(A/C)	0,0119	5998	1	0,9221	T=A,C=C
**rs113610712**	228556727	(-/A)	0,0119	5861	1	0,9221	T=-,C=A
**rs80055005**	228557635	(G/A)	0,0119	4953	1	0,9221	T=G,C=A
**rs74800339**	228557686	(C/T)	0,0119	4902	1	0,9221	T=C,C=T
**rs6728344**	228567046	(T/C)	0,0119	4458	1	0,9221	T=T,C=C
**rs74387373**	228566682	(T/G)	0,0119	4094	1	0,9221	T=T,C=G
**rs75798521**	228558518	(A/G)	0,0119	4070	1	0,9221	T=A,C=G
**rs112219909**	228566330	(T/C)	0,0119	3742	1	0,9221	T=T,C=C
**rs78550805**	228559154	(C/T)	0,0119	3434	1	0,9221	T=C,C=T
**rs111510401**	228559528	(T/C)	0,0119	3060	1	0,9221	T=T,C=C
**rs111856941**	228565218	(T/C)	0,0119	2630	1	0,9221	T=T,C=C
**rs139091057**	228565087	(A/-)	0,0119	2499	1	0,9221	T=A,C=-
**rs6721696**	228560332	(T/C)	0,0119	2256	1	0,9221	T=T,C=C
**rs112560852**	228564718	(T/C)	0,0119	2130	1	0,9221	T=T,C=C
**rs111618899**	228564663	(C/G)	0,0119	2075	1	0,9221	T=C,C=G
**rs34507036**	228560728	(A/G)	0,0119	1860	1	0,9221	T=A,C=G

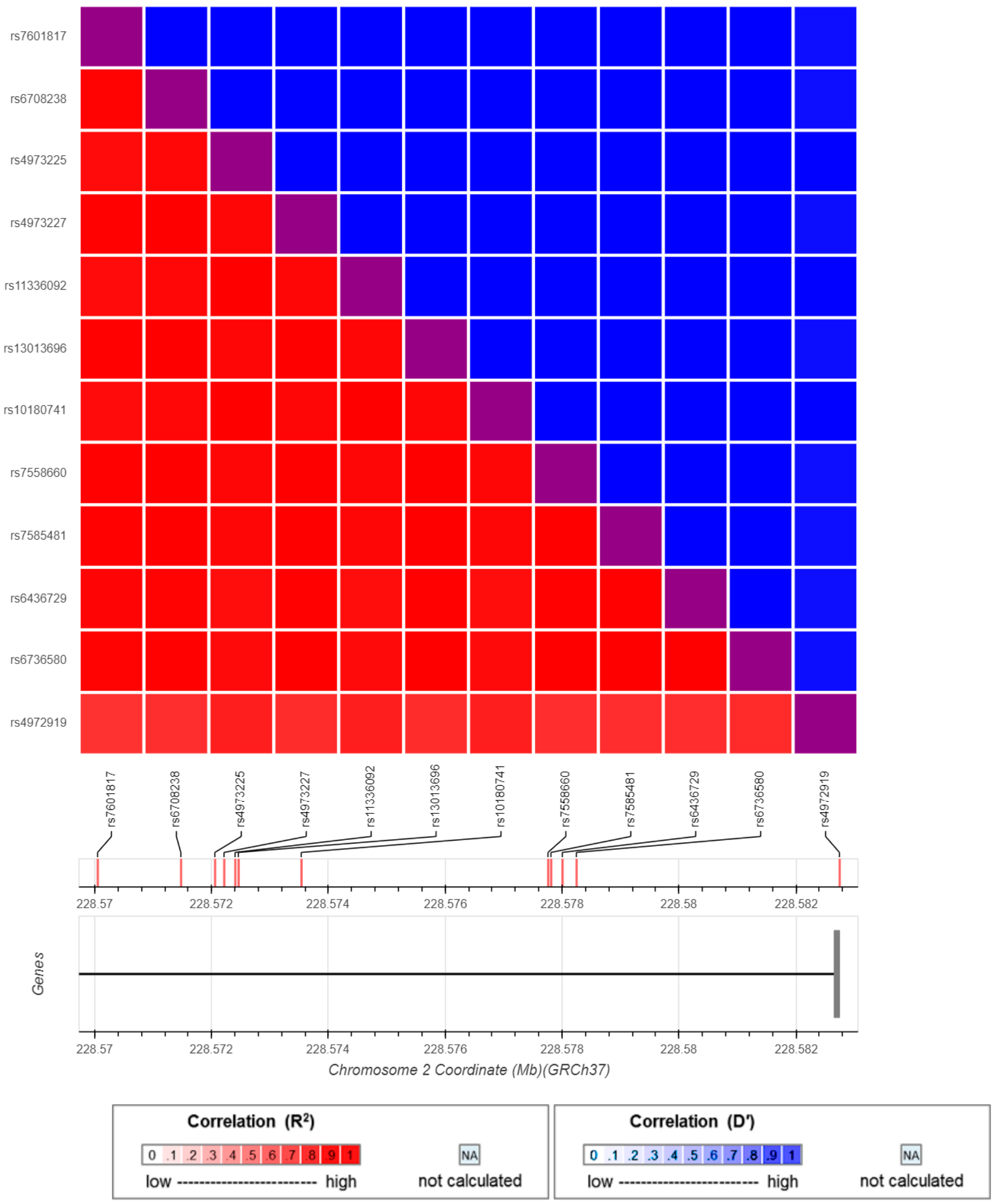

Figure S2. Haplotype 2 Interactive heatmap matrix of Linkage Disequilibrium Calculations: D′ values are represented by the blue squares and R^2 values are represented by the red squares.

Table S2. Characterization of SNPs belonging to the haplotype 2.

RS Results	Position (GRCh37)	Alleles	MAF	Distance	D'	R^2	Correlated Alleles
**rs4973227**	228572214	(A/G)	0,2416	246	1	1	T=A,C=G
**rs7558660**	228577758	(A/G)	0,2425	51	1	1	C=A,T=G
**rs13013696**	228572460	(T/C)	0,2416	5349	1	0,9946	C=T,T=C
**rs7585481**	228577809	(C/T)	0,2425	5349	1	0,9946	T=C,C=T
**rs6708238**	228571475	(C/T)	0,2416	985	0,9946	0,9892	T=T,C=C
**rs6736580**	228578244	(G/A)	0,2425	435	0,9946	0,9892	C=G,T=A
**rs6436729**	228578003	(T/A)	0,2445	194	0,9946	0,9786	C=T,T=A
**rs7601817**	228570050	(A/C)	0,2396	7759	0,9945	0,9731	C=C,T=A
**rs10180741**	228573536	(T/A)	0,2316	1076	1	0,9464	T=A,C=T
**rs11336092**	228572403	(G/-)	0,2316	57	1	0,9464	T=G,C=-
**rs4973225**	228572057	(G/C)	0,2316	403	1	0,9464	T=G,C=C
**rs4972919**	228582746	(G/A)	0,2505	10286	0,9231	0,812	T=G,C=A

Red is the lead SNP.

Table S3. List of transcription factors predicted by bioinformatics methods for SNPs of haplotype 2.

Regions	RS ID	WT	MV
**promoter**	rs4972919	EBF1	ESR1
		EBF2	ESR2
		EBF3	NR2C1
		ETV6	Stat5a
		RELB	THAP1
		RFX4	CUX2
		RREB1	
		SPIB	
		Znf423	
**enhancer5**	rs6436729	Dlx4	EMX1
		FOXB1	EMX2
		LBX2	HMX1
		LHX2	HMX2
		MAFK	HMX3
		MEF2B	HOXA2
		MEF2D	HOXB13
		MSX1	HOXB5
		MSX2	HOXB9
		NR1H4	HOXC10
		NR2C1	HOXC12
		POU1F1	HOXC13
		RORC	HOXD10
		SHOX	HOXD11
		TBP	Lhx4
		Zic2	LHX5
			MEF2A
			MEOX1
			MSX3
			POU4F2
			PROX1
			RAX2
			RFX1
			RFX2
			RFX4
			RFX5
			VAX1
			ZKSCAN1
			ZNF274
			ZNF382
	rs6736580	DLX3	CUX1
		DLX4	CUX2
		EN2	FOXB1
		ESR1	HOXA11
		ESR2	HOXA9
		HMX1	HOXB5
		HMX2	HOXC11
		HMX3	HOXC12
		HOXA13	HOXD10
		HOXB13	HOXD11
		LHX2	LHX4
		MEF2A	MAFK
		MEF2B	POU1F1
		MEF2D	POU4F2
		MSX3	PROX1
		NR2C1	ZNF274
		RAX2	
		RFX3	
		RORC	

WT: wild type, MV: minor variant.

Table S4. Hardy-Weinberg equilibria and minor allele frequencies (MAF) values for the three haplotype variants examined. Results from a total of 262 samples studied.

		Hardy-Weinberg eq. (Chi-square test)	q (MAF)
**Haplotype 2**	rs6436729	0.9784	0.2156
**Haplotype 3**	rs34241868	0.9422	0.0935
**Haplotype 4**	rs55975119	0.6510	0.2080

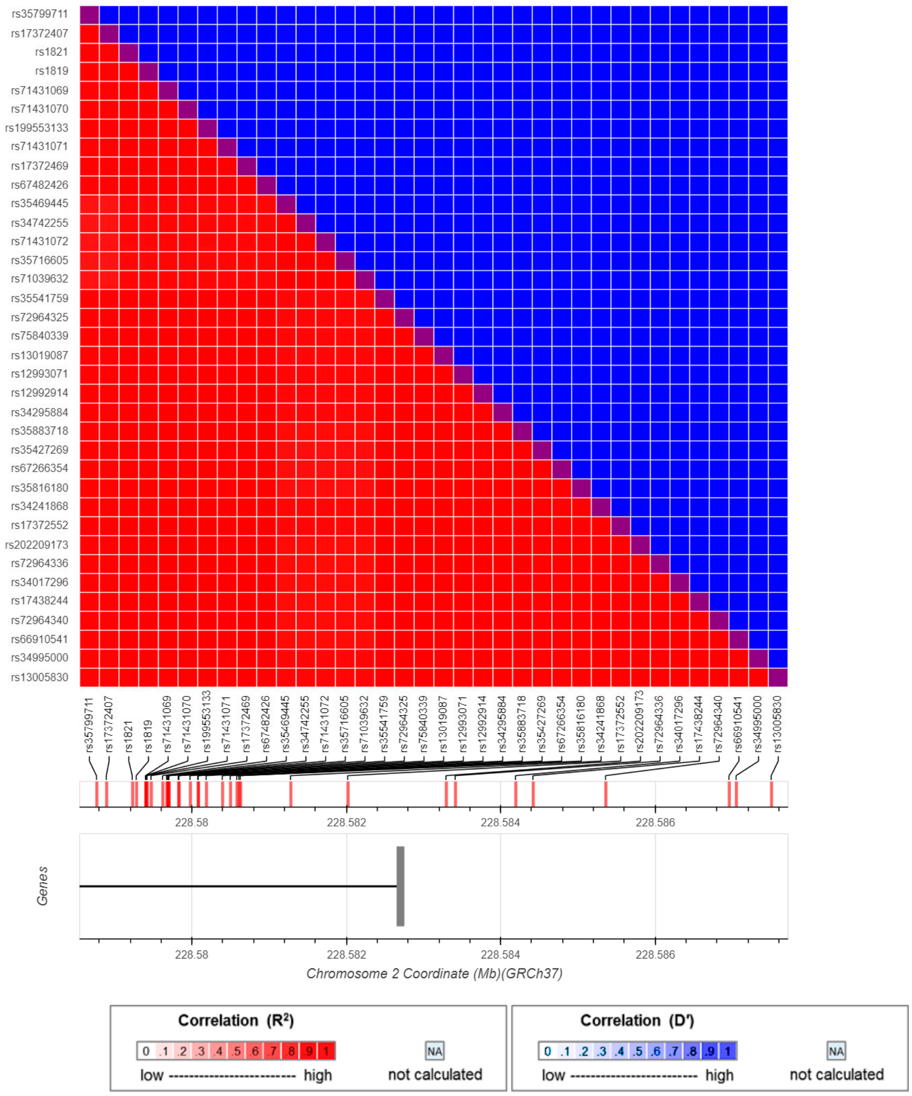

Figure S3. Haplotype 3 Interactive heatmap matrix of Linkage Disequilibrium Calculations: D′ values are represented by the blue squares and R^2 values are represented by the red squares.

Table S5. Characterization of SNPs belonging to the haplotype 3.

RS Results	Position (GRCh37)	Alleles	MAF	Distance	D'	R^2	Correlated Alleles
**rs67482426**	228579619	(A/T)	0,1839	221	1	1	G=A,A=T
**rs71431069**	228579398	(G/A)	0.1839	4	1.0	1.0	A=G,C=A
**rs71431070**	228579402	(A/C)	0,1839	4	1	1	G=A,A=C
**rs71431071**	228579418	(C/T)	0,1839	20	1	1	G=C,A=T
**rs12992914**	228580184	(C/T)	0,1829	786	1	0,9934	G=C,A=T
**rs17372469**	228579470	(T/C)	0,1829	72	1	0,9934	G=T,A=C
**rs17372552**	228582018	(T/C)	0,1829	2620	1	0,9934	G=T,A=C
**rs1819**	228579279	(A/T)	0,1829	119	1	0,9934	G=A,A=T
**rs1821**	228579227	(G/A)	0,1829	171	1	0,9934	G=G,A=A
**rs199553133**	228579404	(T/-)	0,1829	6	1	0,9934	G=T,A=-
**rs34241868**	228581277	(C/A)	0,1829	1879	1	0,9934	G=C,A=A
**rs34295884**	228580393	(G/T)	0,1829	995	1	0,9934	G=G,A=T
**rs35541759**	228579823	(G/A)	0,1829	425	1	0,9934	G=G,A=A
**rs72964325**	228579831	(T/C)	0,1829	433	1	0,9934	G=T,A=C
**rs75840339**	228579975	(A/C)	0,1829	577	1	0,9934	G=A,A=C
**rs12993071**	228580081	(G/T)	0,1819	683	1	0,9868	G=G,A=T
**rs17438244**	228584416	(A/C)	0,1839	5018	0,9934	0,9868	G=A,A=C
**rs202209173**	228583290	(TC/-)	0,1839	3892	0,9934	0,9868	G=TC,A=-
**rs34017296**	228584190	(A/C)	0,1839	4792	0,9934	0,9868	G=A,A=C
**rs35883718**	228580496	(C/T)	0,1819	1098	1	0,9868	G=C,A=T
**rs66910541**	228586955	(T/A)	0,1839	7557	0,9934	0,9868	G=T,A=A
**rs72964336**	228583409	(C/G)	0,1839	4011	0,9934	0,9868	G=C,A=G
**rs72964340**	228585356	(T/C)	0,1839	5958	0,9934	0,9868	G=T,A=C
**rs13005830**	228587502	(C/T)	0,1829	8104	0,9933	0,9802	G=C,A=T
**rs13019087**	228580076	(T/A)	0,1809	678	1	0,9802	G=T,A=A
**rs34995000**	228587047	(G/A)	0,1829	7649	0,9933	0,9802	G=G,A=A
**rs35427269**	228580580	(T/C)	0,1799	1182	1	0,9736	G=T,A=C
**rs35816180**	228580626	(C/A)	0,1799	1228	1	0,9736	G=C,A=A
**rs67266354**	228580607	(G/A)	0,1799	1209	1	0,9736	G=G,A=A
**rs17372407**	228578891	(T/C)	0,1789	507	1	0,9671	G=T,A=C
**rs35799711**	228578764	(T/G)	0,1799	634	0,9932	0,9605	G=T,A=G
**rs34742255**	228579684	(C/T)	0,1799	286	0,9797	0,9345	G=C,A=T
**rs35469445**	228579671	(G/A)	0,1799	273	0,9797	0,9345	G=G,A=A
**rs35716605**	228579693	(G/C)	0,1799	295	0,9797	0,9345	G=G,A=C
**rs71039632**	228579702	(-/T)	0,1799	304	0,9797	0,9345	G=-,A=T
**rs71431072**	228579689	(C/A)	0,1799	291	0,9797	0,9345	G=C,A=A

Red is the lead SNP.

Table S6. List of transcription factors predicted by bioinformatics methods for SNPs of haplotype 3.

Regions	RS ID	WT	MV
**enhancer3**	rs17372552	CUX1	MEF2B
		HOXD10	MEF2D
		Nr5a2	
		NR6A1	
**enhancer4**	rs34241868	KLF13	HES6
		NFYC	RORC
		ZNF382	ZSCAN4

WT: wild type, MV: minor variant.

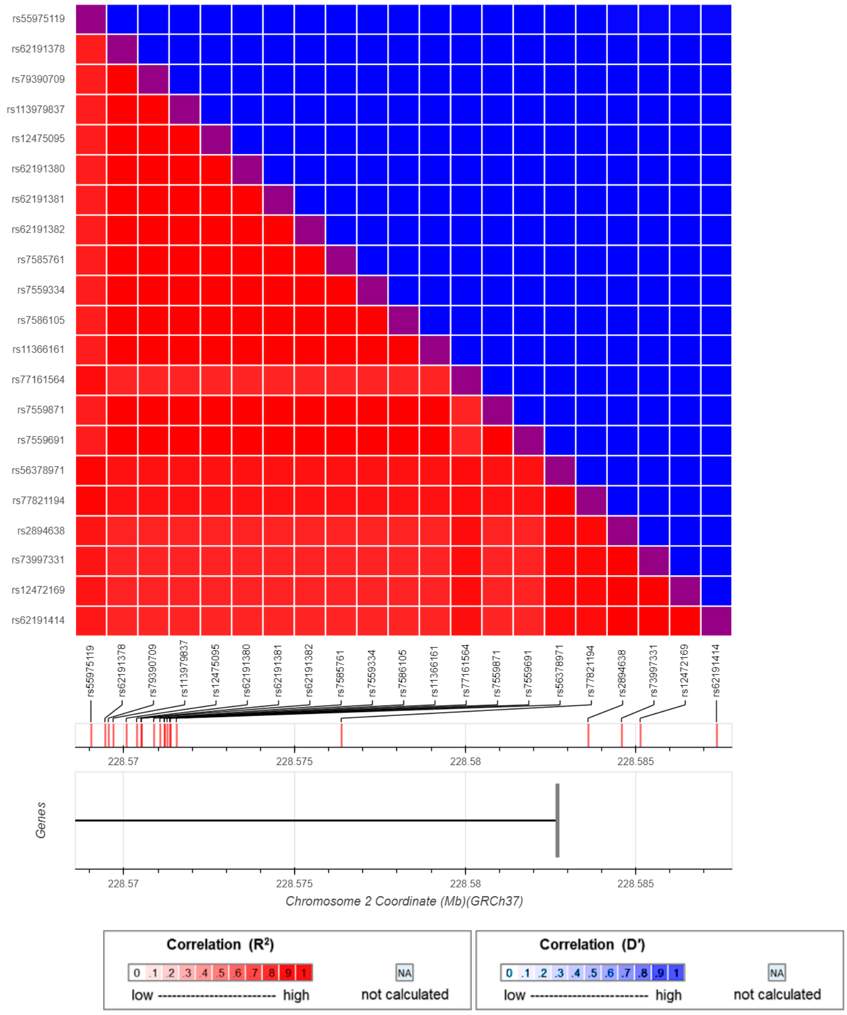

Figure S4. Haplotype 4 Interactive heatmap matrix of Linkage Disequilibrium Calculations: D′ values are represented by the blue squares and R^2 values are represented by the red squares.

Table S7. Characterization of SNPs belonging to the haplotype 4.

RS ID	Position (GRCh37)	Alleles	MAF	Distance	D'	R^2	Correlated Alleles
**rs12472169**	228585123	(C/T)	0,1024	2220	1	1	T=C,C=T
**rs73997331**	228584575	(G/A)	0,1024	1672	1	1	T=G,C=A
**rs2894638**	228583595	(T/C)	0,1024	692	1	1	T=T,C=C
**rs62191414**	228587359	(T/C)	0,1034	4456	1	0,9893	T=T,C=C
**rs56378971**	228571538	(T/C)	0,1064	11365	0,9891	0,9376	T=T,C=C
**rs77821194**	228576366	(C/A)	0,1064	6537	0,9891	0,9376	T=C,C=A
**rs77161564**	228571266	(C/T)	0,0994	11637	0,9777	0,925	T=C,C=T
**rs55975119**	228569035	(C/A)	0,1024	13868	0,9459	0,8948	T=C,C=A
**rs62191378**	228569445	(G/C)	0,1163	13458	0,989	0,8478	T=G,C=C
**rs79390709**	228569547	(C/A)	0,1163	13356	0,989	0,8478	T=C,C=A
**rs113979837**	228569685	(T/C)	0,1163	13218	0,989	0,8478	T=T,C=C
**rs12475095**	228570070	(A/C)	0,1163	12833	0,989	0,8478	T=A,C=C
**rs62191380**	228570375	(T/C)	0,1163	12528	0,989	0,8478	T=T,C=C
**rs62191381**	228570502	(C/T)	0,1163	12401	0,989	0,8478	T=C,C=T
**rs62191382**	228570515	(C/T)	0,1163	12388	0,989	0,8478	T=C,C=T
**rs7585761**	228570875	(A/G)	0,1163	12028	0,989	0,8478	T=A,C=G
**rs7559334**	228571056	(C/T)	0,1163	11847	0,989	0,8478	T=C,C=T
**rs7586105**	228571182	(A/C)	0,1163	11721	0,989	0,8478	T=A,C=C
**rs11366161**	228571189	(T/-)	0,1163	11714	0,989	0,8478	T=T,C=-
**rs7559871**	228571344	(G/A)	0,1163	11559	0,989	0,8478	T=G,C=A
**rs7559691**	228571361	(C/T)	0,1163	11542	0,989	0,8478	T=C,C=T

Red is the lead SNP.

Table S8. List of transcription factors predicted by bioinformatics methods for SNPs of haplotype 4.

Regions	RS ID	WT	MV
**enhancer9**	rs55975119	ERG	Ar
		ETS2	DLX4
		ETV2	EN2
		FLI1	ESR2
		HOXA9	LHX2
		HOXC12	NR1I2
		HOXD11	NR3C1
		LHX4	NR3C2
		POU4F2	STAT1
**enhancer8**	rs62191380	RFX1	RFX3
		RFX5	
		ZNF274	
	rs62191382	A6NFQ7	
		Ddit3	
		GCM1	
		GSC	
		MEIS1	
		PAX9	
		PROP1	
		ZIC3	
		ZIC5	
		ZNF140	
		ZNF143	
		ZNF682	
**enhancer1**	rs73997331	EBF1	Arid5a
		ETS2	OLIG2
		PROX1	OLIG3
**enhancer7**	rs7559334	ARID3B	GSC
		Esrrg	IRF9
		GATA1	Smad4
		HSF1	
		HSF4	
		Lhx3	
		NR2F1	
		OTX1	
		PITX1	
		RELB	
	rs7585761	RFX2	

WT: wild type, MV: minor variant.

Table S9. General characteristics and laboratory dates of control and type 2 diabetes groups. (Mean±SD).

	CTRL	T2DM	p
**n**	54	66	-
**age (years)**	62.3±10.6	63.5±8.6	**0.5082** [a]
**male (n)**	29	25	
**female (n)**	25	41	
**height (cm)**	169.4±11.79	167.4±11.68	**0.3511** [a]
**weight (kg)**	75.42±19.7	82.28±19.27	**0.0574** [a]
**abdominal circumference (cm)**	94.71±12.15	101.5±11.32	**0.0020** [a]
**BMI (kg/m²)**	26.78±4.345	30.01±4.64	**0.0002** [a]
**blood glucose (mmol/L)**	5.017±0.4693	6.489±2.143	**<0.0001** [b]
**blood insulin (mIU/L)**	7.746±4.721	9.998±4.791	**0.0023** [b]
**HOMA**	1.833±1.27	2.985±1.849	**<0.0001** [b]
**HgA1c (%)**	5.32±0.4939	6.521±1.192	**<0.0001** [b]
**uric acid (µmol/L)**	319±67.96	339.9±88.63	**0.2254** [b]
**RBC (tera/L)**	4.662±0.3725	4.862±0.3879	**0.0098** [b]
**WBC (giga/L)**	5.98±1.61	7.136±2.276	**0.0028** [b]
**neutrophil (%)**	57.76±7.58	58±10.06	**0.4984** [b]
**lympho (%)**	30.65±9.805	29.94±6.71	**0.7336** [b]
**mono (%)**	6.58±1.499	6.159±1.285	**0.0846** [b]
**eosinophil (%)**	2.98±1.842	2.75±1.62	**0.5781** [b]
**basophil (%)**	0.5889±0.3368	0.5348±0.2421	**0.6392** [b]
**platelet (giga/L)**	239.6±59.07	244.2±51.59	**0.6371** [b]

The statistical analysis of physical parameters was Student t-test (a), the statistical analysis of laboratory parameters was Mann-Whitney U test (b). Red is $p < 0.05$. BMI: Body Mass Index, HOMA: Homeostasis Model Assessment, HgA1c: Hemoglobin A1C, RBC: red blood cells, WBC: white blood cells.

Table S10. The occurrence of the SNP rs6436729 (Haplotype 2) in the healthy and T2DM populations.

	n				Hardy-Weinberg eq. (Chi-square test)	q (MAF)
	all	WT	hetero	homo		
**CTLR**	54	34	19	1	0.7814	0.1944
**T2DM**	66	39	21	6	0.7044	0.25

Table S11. Statistical analysis of the correlation of physical and laboratory parameters and the presence of the rs6436729 (haplotype 2) variant in the groups of control individuals and T2DM patients.

A	CTRL WT	CTRL MV	T2DM WT	T2DM MV	p
**n**	34	20	39	27	-
**height (cm)**	168.4±12.16	171.1±11.21	168±11.06	166.5±12.68	**0.6151** [a]
**weight (kg)**	76.11±19.41	74.26±20.64	81.47±20.03	83.44±18.41	**0.2777** [a]
**abdominal circumference (cm)**	94.04±13.87	95.85±8.70	101.1±10.3	102.1±12.83	**0.02** [a]
**BMI (kg/m²)**	26.48±4.301	27.3±4.48	29.85±4.84	30.23±4.41	**0.0022** [a]
**blood glucose (mmol/L)**	5.04±0.47	4.98±0.48	6.76±2.53	6.1±1.35	**<0.0001** [b]
**blood insulin (mIU/L)**	7.31±4.23	8.5±5.5	9.84±3.90	10.23±5.92	**0.0202** [b]
**HOMA**	1.68±1.09	2.09±1.53	3.03±1.77	2.92±1.99	**0.0002** [b]
**HgA1c (%)**	5.3±0.6	5.36±0.25	6.54±1.18	6.5±1.23	**<0.0001** [b]
**uric acid (μmol/L)**	317.4±74.66	321.7±56.47	354.8±80.85	318.5±96.33	**0.1372** [b]
**RBC (tera/L)**	4.61±0.35	4.75±0.40	4.88±0.41	4.83±0.35	**0.0478** [b]
**WBC (giga/L)**	5.99±1.69	5.97±1.51	6.82±2.02	7.59±2.57	**0.0163** [b]
**neutrophil (%)**	57.58±7.32	58.07±8.18	58.08±7.41	57.87±13.14	**0.7336** [b]
**lympho (%)**	30.92±11.18	30.2±7.12	30.49±6.6	29.14±6.93	**0.891** [b]
**mono (%)**	6.57±1.62	6.59±1.62	6.15±1.167	6.17±1.46	**0.3885** [b]
**eosinophil (%)**	3.08±1.84	2.81±1.88	2.83±1.83	2.64±1.28	**0.7595** [b]
**basophil (%)**	0.64±0.37	0.5±0.24	0.54±0.25	0.53±0.23	**0.442** [b]
**platelet (giga/L)**	242.2±65.98	235.3±46.27	247.2±49.53	240±55.11	**0.8855** [b]

Summary of physical and laboratory parameters for age-matched control subjects and the T2DM patients (Mean±SD). The statistical analysis of physical parameters was ANOVA (a), the statistical analysis of laboratory parameters was Kruskal-Wallis test (b).

B	Significance of differences	CTRL MV	T2DM MV	CTRL WT
**abdominal circumference** [a]	CTRL WT	**0.6027**	0.0238	-
	T2DM WT	0.0573	**0.7278**	0.0155
	T2DM MV	0.0677	-	-
**BMI** [a]	CTRL WT	**0.5113**	0.0015	-
	T2DM WT	0.0540	**0.7492**	0.0026
	T2DM MV	0.0302	-	-
**blood glucose** [b]	CTRL WT	**0.6655**	0.0009	-
	T2DM WT	<0.0001	**0.5705**	<0.0001
	T2DM MV	0.0032	-	-
**blood insulin** [b]	CTRL WT	**0.591**	0.0449	-
	T2DM WT	0.0658	**0.7981**	0.0023
	T2DM MV	0.2972	-	-
**HOMA** [b]	CTRL WT	**0.513**	0.0058	-
	T2DM WT	0.0087	**0.6019**	<0.0001
	T2DM MV	0.1258	-	-
**HgA1c** [b]	CTRL WT	**0.975**	<0.0001	-
	T2DM WT	<0.0001	**0.894**	<0.0001
	T2DM MV	<0.0001	-	-
**uric acid** [b]	CTRL WT	**0.8279**	0.787	-
	T2DM WT	0.1199	**0.0633**	0.0536
	T2DM MV	0.6353	-	-
**RBC** [b]	CTRL WT	**0.357**	0.0403	-
	T2DM WT	0.2733	**0.6808**	0.0063
	T2DM MV	0.4914	-	-
**WBC** [b]	CTRL WT	**0.9894**	0.0049	-
	T2DM WT	0.1635	**0.233**	0.0608
	T2DM MV	0.0129	-	-

Two component statistical analysis of the correlation of the significant laboratory parameters and the presence of the rs6436729 variant in the groups of control individuals and T2DM patients. The statistical analysis was Student t-test (a), or Mann Whitney U-test (b). Red is $p < 0.05$. BMI: Body Mass Index, HOMA: Homeostasis Model Assessment, HgA1c: Hemoglobin A1C, RBC: red blood cells, WBC: white blood cells.

Table S12. The occurrence of the SNP rs34241868 (Haplotype 3) in the healthy and T2DM populations.

	**n**				**Hardy-Weinberg eq. (Chi-square test)**	**q (MAF)**
	all	WT	hetero	homo		
**CTLR**	54	36	17	1	0.8913	0.1759
**T2DM**	66	40	25	1	0.5684	0.2045

Table S13. Statistical analysis of the correlation of physical and laboratory parameters and the presence of the rs34241868 variant in the groups of control individuals and T2DM patients.

A	**CTRL WT**	**CTRL MV**	**T2DM WT**	**T2DM MV**	**p**
**n**	36	18	40	26	-
**height (cm)**	170.1±11.91	167.9±11.72	169.1±11.87	164.7±11.08	**0.3287** [a]
**weight (kg)**	76.78±22.03	72.7±14.09	82.6±21.17	81.78±16.29	**0.2477** [a]
**abdominal circumference (cm)**	97.43±12.31	89.28±10.08	101.8±11.38	101±11.42	**0.0015** [a]
**BMI (kg/m^2)**	27.33±4.853	25.68±2.91	30±4.73	30.01±4.59	**0.0013** [a]
**blood glucose (mmol/L)**	4.99±0.47	5.08±0.48	6.02±1.37	7.21±2.85	**<0.0001** [b]
**blood insulin (mIU/L)**	7.96±4.62	7.31±5.02	9.03±4.46	11.45±5.01	**0.0047** [b]
**HOMA**	1.81±1.17	1.87±1.48	2.50±1.84	3.73±2.19	**<0.0001** [b]
**HgA1c (%)**	5.4±0.21	5.17±0.79	6.20±0.65	7.01±1.62	**<0.0001** [b]
**uric acid (µmol/L)**	326.9±69.68	303.1±63.26	332.3±86.59	351.8±92.14	**0.3083** [b]
**RBC (tera/L)**	4.71±0.34	4.56±0.43	4.90±0.41	4.80±0.35	**0.0507** [b]
**WBC (giga/L)**	5.89±1.62	6.15±1.62	7.07±2.6	7.24±1.63	**0.0116** [b]
**neutrophil (%)**	57.84±7.97	57.61±6.95	58.99±8.49	56.47±12.1	**0.8319** [b]
**lympho (%)**	30.93±11.33	30.1±5.89	29.45±7.43	30.68±5.47	**0.8515** [b]
**mono (%)**	6.43±1.56	6.88±1.36	6.32±1.38	5.92±1.11	**0.1916** [b]
**eosinophil (%)**	2.91±1.56	3.13±2.35	2.77±1.38	2.72±1.97	**0.7898** [b]
**basophil (%)**	0.64±0.35	0.48±0.3	0.55±0.26	0.51±0.22	**0.1124** [b]
**platelet (giga/L)**	240.8±63.46	237.2±50.78	242.1±53.57	247.5±46.24	**0.9397** [b]

Summary of physical and laboratory parameters for age-matched control subjects and the T2DM patients (Mean±SD). The statistical analysis of physical parameters was ANOVA (a), the statistical analysis of laboratory parameters was Kruskal-Wallis test (b).

B	Significance of differences	CTRL MV	T2DM MV	CTRL WT
**abdominal circumference** [a]	CTRL WT	**0.0186**	0.2554	-
	T2DM WT	0.0002	**0.7646**	0.1102
	T2DM MV	0.0011	-	-
**BMI** [a]	CTRL WT	**0.1907**	0.0321	-
	T2DM WT	0.0007	**0.9919**	0.0177
	T2DM MV	0.0010	-	-
**blood glucose** [b]	CTRL WT	**0.4399**	<0.0001	-
	T2DM WT	0.0047	**0.0756**	<0.0001
	T2DM MV	<0.0001	-	-
**blood insulin** [b]	CTRL WT	**0.3794**	0.0041	-
	T2DM WT	0.05	**0.0571**	0.1698
	T2DM MV	0.002	-	-
**HOMA** [b]	CTRL WT	**0.5942**	<0.0001	-
	T2DM WT	0.0343	**0.0189**	0.0088
	T2DM MV	0.0005	-	-
**HgA1c** [b]	CTRL WT	**0.2776**	<0.0001	-
	T2DM WT	<0.0001	**0.0102**	<0.0001
	T2DM MV	<0.0001	-	-
**uric acid** [b]	CTRL WT	**0.2336**	0.215	-
	T2DM WT	0.3224	**0.3455**	0.9279
	T2DM MV	0.0762	-	-
**RBC** [b]	CTRL WT	**0.3944**	0.3117	-
	T2DM WT	0.015	**0.5683**	0.0433
	T2DM MV	0.0613	-	-
**WBC** [b]	CTRL WT	**0.6264**	0.0009	-
	T2DM WT	0.2607	**0.2079**	0.0688
	T2DM MV	0.0265	-	-

Two component statistical analysis of the correlation of the significant laboratory parameters and the presence of the rs34241868 variant in the groups of control individuals and T2DM patients. The statistical analysis was Student t-test (a), or Mann Whitney U-test (b). Red is $p < 0.05$. BMI: Body Mass Index, HOMA: Homeostasis Model Assessment, HgA1c: Hemoglobin A1C, RBC: red blood cells, WBC: white blood cells.

Table S14. Statistical analysis of the correlation between HOMA or HbA1c (%) and the presence of the Haplotype 3 minor variant in groups of healthy individuals and T2DM patients, respectively.

		WT	MV	p
**HOMA**	CTRL	1.81±1.17 (36)	1.87±1.48 (18)	0.5942
	T2DM	2.50±1.84 (40)	3.73±2.19 (26)	0.0189
**HbA1c (%)**	CTRL	5.4±0.21 (36)	5.17±0.79 (18)	0.2776
	T2DM	6.20±0.65 (40)	7.01±1.62(26)	0.0102

Values are expressed mean±SD (n). The p values were calculated by the Mann Whitney U-test, red p level is $p<0.05$.

Table S15. The occurrence of the SNP rs55975119 (Haplotype 4) in the control and T2DM populations.

	n				Hardy-Weinberg eq. (Chi-square test)	q (MAF)
	all	WT	hetero	homo		
**CTLR**	54	44	9	1	0.9198	0.1018
**T2DM**	66	52	14	0	0.6601	0.106

Table S16. Statistical analysis of the correlation of physical and laboratory parameters and the presence of the rs55975119 variant in the groups of control individuals and T2DM patients.

A	CTRL WT	CTRL MV	T2DM WT	T2DM MV	p
**n**	44	10	52	14	-
**height (cm)** [a]	168.5±12	173.4±10.39	168.2±11.47	164.1±12.3	**0.3026**
**weight (kg)** [a]	74.42±20.63	79.85±15.02	82.71±19.27	80.66±19.88	**0.2279**
**abdominal circumference (cm)** [a]	94.42±13.04	96±7.47	101.8±10.74	100.3±13.62	**0.0207**
**BMI (kg/m²)** [a]	26.73±4.66	27±2.72	30.15±4.75	29.47±4.31	**0.0024**
**blood glucose (mmol/L)** [b]	5.08±0.46	4.74±0.43	6.54±2.31	6.31±1.41	**<0.0001**
**blood insulin (mIU/L)** [b]	7.81±4.92	7.48±3.9	10.04±4.31	9.85±6.49	**0.0188**
**HOMA** [b]	1.82±1.28	1.89±1.27	3.03±1.82	2.83±2.01	**0.0003**
**HgA1c (%)** [b]	5.31±0.53	5.38±0.26	6.489±1.09	6.68±1.56	**<0.0001**
**uric acid (µmol/L)** [b]	314.1±68.5	340.3±64.49	357.8±85.67	273.8±67.14	**0.0025**
**RBC (tera/L)** [b]	4.61±0.36	4.89±0.37	4.89±0.387	4.74±0.382	**0.006**
**WBC (giga/L)** [b]	6±1.7	5.9±1.22	6.72±1.85	8.66±3.05	**0.0045**
**neutrophil (%)** [b]	57.3±7.94	59.82±5.6	57.15±10.05	61.15±9.77	**0.396**
**lympho (%)** [b]	31.27±10.57	27.96±4.79	30.26±6.29	28.75±8.25	**0.6686**
**mono (%)** [b]	6.46±1.50	7.13±1.42	6.26±1.31	5.79±1.17	**0.1269**
**eosinophil (%)** [b]	3.03±1.88	2.75±1.76	2.85±1.74	2.38±1.03	**0.834**
**basophil (%)** [b]	0.61±0.35	0.5±0.27	0.56±0.25	0.45±0.21	**0.3578**
**platelet (giga/L)** [b]	242.9±62.68	225.2±38.63	240.2±50.97	259.3±52.95	**0.3988**

Summary of physical and laboratory parameters for age-matched control subjects and the T2DM patients (Mean±SD). The statistical analysis of physical parameters was ANOVA (a), the statistical analysis of laboratory parameters was Kruskal-Wallis test (b).

B	Significance of differences	CTRL MV	T2DM MV	CTRL WT
**abdominal circumference** [a]	CTRL WT	**0.7144**	0.1523	-
	T2DM WT	0.1083	**0.6586**	0.0030
	T2DM MV	0.3781	-	-
**BMI** [a]	CTRL WT	**0.8611**	0.0568	-
	T2DM WT	0.0476	**0.6280**	0.0006
	T2DM MV	0.1260	-	-
**blood glucose** [b]	CTRL WT	**0.0474**	0.0008	-
	T2DM WT	<0.0001	**0.7889**	<0.0001
	T2DM MV	0.0035	-	-
**blood insulin** [b]	CTRL WT	**0.9432**	0.3798	-
	T2DM WT	0.0678	**0.5016**	0.0019
	T2DM MV	0.4811	-	-
**HOMA** [b]	CTRL WT	**0.8649**	0.0624	-
	T2DM WT	0.0342	**0.5267**	<0.0001
	T2DM MV	0.2286	-	-
**HgA1c** [b]	CTRL WT	**0.748**	<0.0001	-
	T2DM WT	<0.0001	**0.9534**	<0.0001
	T2DM MV	<0.0001	-	-
**uric acid** [b]	CTRL WT	**0.3242**	0.0541	-
	T2DM WT	0.0358	**0.0008**	0.0113
	T2DM MV	0.5807	-	-
**RBC** [b]	CTRL WT	**0.0496**	0.3749	-
	T2DM WT	0.9287	**0.168**	0.0006
	T2DM MV	0.3948	-	-
**WBC** [b]	CTRL WT	**0.9345**	0.0012	-
	T2DM WT	0.1782	**0.0272**	0.0429
	T2DM MV	0.0093	-	-

Two component statistical analysis of the correlation of the significant laboratory parameters and the presence of the rs55975119 variant in the groups of control individuals and T2DM patients. The statistical analysis was Student t-test (a), or Mann Whitney U-test (b). Red is $p < 0.05$. BMI: Body Mass Index, HOMA: Homeostasis Model Assessment, HgA1c: Hemoglobin A1C, RBC: red blood cells, WBC: white blood cells.

Table S17. Statistical analysis of the correlation between uric acid and WBC count and the presence of the Haplotype 4 minor variant in groups of healthy individuals and T2DM patients.

		WT	MV	p
**uric acid (µmol/L)**	CTRL	314.1±68.5 (44)	340.3±64.49 (10)	0.3242
	T2DM	357.8±85.67 (52)	273.8±67.14 (14)	0.0008
**WBC (giga/L)**	CTRL	6±1.7 (44)	5.9±1.22 (10)	0.9345
	T2DM	6.72±1.85 (52)	8.66±3.05 (14)	0.0272

Mean±SD (n), the p values were calculated by the Mann Whitney U-test, red p level is $p<0.05$.

Table S18. General laboratory dates of control and gout groups. (Mean±SD).

	**n CTRL**	**n AU**	**CTRL**	**AU**	**p**
**age**	73	76	55±13.3	57.4±11.3	**0.233**
**uric acid (µmol/L)**	66	75	320±57.88	469.8±117	**<0.0001**
**RBC (tera/L)**	65	72	4.87±0.41	4.78±0.51	**0.5785**
**WBC (giga/L)**	65	72	8.21±3.07	8.16±2.77	**0.9358**
**neutrophil (giga/L)**	61	67	5.29±2.74	6.92±9.55	**0.5154**
**lympho (giga/L)**	61	67	2.17±0.87	3.02±5.27	**0.982**
**mono (giga/L)**	61	67	0.46±0.15	0.60±0.98	**0.9953**
**eosinophil (giga/L)**	61	67	0.17±0.10	0.22±0.26	**0.5416**
**basophil (giga/L)**	61	67	0.04±0.02	0.06±0.10	**0.0977**
**neutrophil %**	62	69	62.11±9.86	60.79±14.41	**0.9519**
**lympho %**	62	69	27.75±8.25	25.91±9.92	**0.3488**
**mono%**	62	69	5.7±1.51	5.27±1.87	**0.2092**
**eosinophil %**	62	69	2.37±1.61	2.33±1.83	**0.64**
**basophil %**	61	69	0.51±0.22	0.63±0.53	**0.4857**
**ESR (mm/h)**	65	68	8.03±11.35	18.72±22.93	**0.0001**
**urea (U/L)**	67	74	5.70±1.5	6.02±2.38	**0.9254**
**creatinine (U/L)**	68	74	86.22±16.66	92.13±24.43	**0.2117**

The statistical analysis of laboratory parameters was Mann-Whitney U test. Red is $p < 0.05$. RBC: red blood cells, WBC: white blood cells, ESR: erythrocyte sedimentation rate.

Table S19. The occurrence of the SNP rs6436729 (Haplotype 2) in the control and gout populations.

	**n**				**Hardy-Weinberg eq. (Chi-square test)**	**q (MAF)**
	all	WT	hetero	homo		
**CTLR**	73	48	22	3	0.9864	0.1918
**AU**	76	47	26	3	0.9835	0.2105

Table S20. Statistical analysis of the correlation of laboratory parameters and the presence of the rs6436729 variant in the groups of control individuals and gouty patients.

A	CTRL WT	CTRL MV	AU WT	AU MV	p
**uric acid (μmol/L)**	324.10±58.22 (45)	311.30±57.57 (21)	485.1±121 (46)	445.5±108.1 (29)	**<0.0001**
**RBC (tera/L)**	4.84±0.44 (43)	4.92±0.36 (22)	4.76±0.51 (46)	4.83±0.51 (26)	**0.5008**
**WBC (giga/L)**	7.78±2.82 (43)	9.06±3.40 (22)	8.26±2.99 (46)	7.97±2.36 (26)	**0.3983**
**neutrophil (giga/L)**	4.73±2.10 (40)	6.35±3.49 (21)	7.74±11.49 (45)	5.25±2.27 (22)	**0.1737**
**lympho (giga/L)**	2.21±0.95 (40)	2.09±0.71 (21)	3.43±6.40 (45)	2.19±0.74 (22)	**0.9782**
**mono (giga/L)**	0.44±0.16 (40)	0.47±0.14 (21)	0.70±1.19 (45)	0.41±0.10 (22)	**0.6578**
**eosinophil (giga/L)**	0.17±0.10 (40)	0.15±0.11 (21)	0.23±0.31 (45)	0.19±0.11 (22)	**0.7268**
**basophil (giga/L)**	0.04±0.02 (40)	0.04±0.02 (21)	0.07±0.13 (45)	0.05±0.02 (22)	**0.4209**
**neutrophil %**	60.26±7.95 (40)	65.47±12.11 (22)	60.44±15.76 (46)	61.50±11.56 (22)	**0.5475**
**lympho %**	29.13±6.97 (40)	25.23±9.86 (22)	24.49±9.80 (46)	28.75±9.73 (23)	**0.1079**
**mono%**	5.82±1.39 (40)	5.48±1.73 (22)	5.27±1.99 (46)	5.28±1.66 (23)	**0.4991**
**eosinophil %**	2.54±1.65 (40)	2.06±1.52 (22)	2.22±1.95 (46)	2.56±1.56 (23)	**0.3802**
**basophil %**	0.54±0.20 (40)	0.46±0.26 (22)	0.66±0.61 (46)	0.57±0.32 (23)	**0.7018**
**ESR (mm/h)**	9.56±13.31 (43)	5.05±4.91 (22)	21.16±24.88 (44)	14.25±18.49 (24)	**0.0002**
**urea (U/L)**	5.596±1.51 (45)	5.91±1.50 (22)	6.18±2.67 (45)	5.76±1.85 (29)	**0.8880**
**creatinine (U/L)**	87.78±16.83 (45)	83.17±16.25 (23)	94.10 ±27.36 (45)	89.06±19.05 (29)	**0.3902**

Summary parameters for age-matched control subjects and the gout patients (Mean±SD (n)). The statistical analysis of laboratory parameters was Kruskal-Wallis test

B		CTRL MV	AU MV	CTRL WT
**ESR**	CTRL WT	**0.0113**	0.1562	-
	AU WT	<0.0001	**0.3901**	0.0059
	AU MV	0.0047	-	-
**uric acid**	CTRL WT	**0.171**	<0.0001	-
	AU WT	<0.0001	**0.1623**	<0.0001
	AU MV	<0.0001	-	-

Two component statistical analysis of the correlation of the significant laboratory parameters and the presence of the rs6436729 variant in the groups of control individuals and AU patients. The statistical analysis was Mann Whitney U-test. Red is $p < 0.05$. RBC: red blood cells, WBC: white blood cells, ESR: erythrocyte sedimentation rate.

Table S21. The occurrence of the SNP rs34241868 (Haplotype 3) in the control and gout populations.

	n				Hardy-Weinberg eq. (Chi-square test)	q (MAF)
	all	WT	hetero	homo		
**CTLR**	73	46	25	2	0.8908	0.1986
**AU**	76	41	30	5	0.9939	0.2632

Table S22. Statistical analysis of the correlation of laboratory parameters and the presence of the rs34241868 variant in the groups of control individuals and gouty patients.

A	CTRL WT	CTRL MV	AU WT	AU MV	p
**uric acid (µmol/L)**	321.20±54 (43)	317.90±65.77 (23)	462.20±116.30 (41)	478.9±119.1 (34)	**<0.0001**
**RBC (tera/L)**	4.91±0.47 (42)	4.79±0.28 (23)	4.87±0.45 (39)	4.68±0.56 (33)	**0.2319**
**WBC (giga/L)**	8.36±3.23 (42)	7.94±2.78 (23)	8.17±2.66 (39)	8.14±2.93 (33)	**0.9343**
**neutrophil (giga/L)**	5.46±3.04 (40)	4.97±2.09 (21)	8..23±12.75 (36)	5.40±2.49 (31)	**0.8451**
**lympho (giga/L)**	2.15±0.78 (40)	2.20±1.05 (21)	3.87±7.09 (36)	2.03±0.81 (31)	**0.5187**
**mono (giga/L)**	0.46±0.14 (40)	0.43±0.17 (21)	0.76±1.32 (36)	0.43±0.14 (31)	**0.3777**
**eosinophil (giga/L)**	0.17±0.10 (40)	0.16±0.11 (21)	0.23±0.33 (36)	0.21±0.14 (31)	**0.4681**
**basophil (giga/L)**	0.04±0.02 (40)	0.04±0.03 (21)	0.07±0.14 (36)	0.05±0.03 (31)	**0.1612**
**neutrophil %**	62.18±10.69 (41)	61.96±8.23 (21)	58.79±16.08 (38)	63.25±11.86 (31)	**0.5948**
**lympho %**	27.59±8.95 (41)	28.06±6.89 (21)	26.69±10.59 (38)	24.95±9.10 (31)	**0.4357**
**mono%**	5.78±1.56 (41)	5.54±1.43 (21)	5.23±1.95 (38)	5.34±1.80 (31)	**0.4419**
**eosinophil %**	2.43±1.57 (41)	2.24±1.71 (21)	2.04±1.49 (38)	2.69±2.15 (31)	**0.5488**
**basophil %**	0.49±0.22 (40)	0.54±0.24 (21)	0.54±0.48 (38)	0.73±0.57 (31)	**0.0797**
**ESR (mm/h)**	8.98±13.68 (43)	6.18±3.54 (22)	14.74±17.82 (38)	23.77±27.61 (30)	**0.0009**
**urea (U/L)**	5.56±1.29 (44)	5.96±1.86 (23)	5.98±1.95 (40)	6.06±2.83 (34)	**0.7997**
**creatinine (U/L)**	86.52±14.59 (45)	85.64±20.48 (23)	92.22 ±21.22 (40)	92.02±28.07 (34)	**0.4655**

Summary parameters for age-matched control subjects and the gout patients (Mean±SD (n)). The statistical analysis of laboratory parameters was Kruskal-Wallis test

B		CTRL MV	AU MV	CTRL WT
**ESR**	CTRL WT	**0.7315**	0.0002	-
	AU WT	0.0648	**0.1641**	0.0357
	AU MV	0.0011	-	-
**uric acid**	CTRL WT	**0.6329**	<0.0001	-
	AU WT	<0.0001	**0.9178**	<0.0001
	AU MV	<0.0001	-	-
**basophil %**	CTRL WT	**0.4651**	0.0236	-
	AU WT	0.3691	**0.0273**	0.5929
	AU MV	0.2123	-	-

Two component statistical analysis of the correlation of the significant laboratory parameters and the presence of the rs34241868 variant in the groups of control individuals and AU patients. The statistical analysis was Mann Whitney U-test. Red is p < 0.05. RBC: red blood cells, WBC: white blood cells, ESR: erythrocyte sedimentation rate.

Table S23. Statistical analysis of the correlation between basophil% and the presence of the Haplotype 3 minor variant in groups of healthy individuals and AU patients. Mean±SD (n).

		WT	MV	P
**basophil%**	CTRL	0.49±0.22 (40)	0.54±0.24 (21)	0.4651
	AU	0.54±0.48 (38)	0.73±0.57 (31)	0.0273

The p values were calculated by Mann Whitney U-test, red p level is p<0.05.

Table S24. The occurrence of the SNP rs55975119 (Haplotype 4) in the control and gout populations.

	n				Hardy-Weinberg eq. (Chi-square test)	q (MAF)
	all	WT	hetero	homo		
**CTLR**	73	60	13	0	0.7278	0.089
**AU**	76	67	7	2	0.4304	0.0724

Table S25. Statistical analysis of the correlation of laboratory parameters and the presence of the rs55975119 variant in the groups of control individuals and gouty patients

A	CTRL WT	CTRL MV	AU WT	AU MV	p
**uric acid (µmol/L)**	323.20±57.6 (55)	304.10±56.72 (11)	463±112 (66)	519.6±147 (9)	**<0.0001**
**RBC (tera/L)**	4.86±0.41 (55)	4.94±0.42 (10)	4.76±0.52 (63)	4.93±0.41 (9)	**0.5718**
**WBC (giga/L)**	7.96±3.05 (55)	9.62±2.88 (10)	8.23±2.87 (63)	7.64±1.92 (9)	**0.2634**
**neutrophil (giga/L)**	4.97±2.58 (51)	6.93±3.07 (10)	7.27±10.21 (58)	4.65±1.69 (9)	**0.096**
**lympho (giga/L)**	2.21±0.91 (51)	1.93±0.64 (10)	3.14±5.66 (58)	2.25±0.77 (9)	**0.8721**
**mono (giga/L)**	0.45±0.15 (51)	0.46±0.16 (10)	0.64±1.05 (58)	0.39±0.12 (9)	**0.8021**
**eosinophil (giga/L)**	0.17±0.10 (51)	0.13±0.13 (10)	0.22±0.27 (58)	0.21±0.15 (9)	**0.2857**
**basophil (giga/L)**	0.04±0.02 (51)	0.04±0.03 (10)	0.06±0.11 (58)	0.04±0.02 (9)	**0.3945**
**neutrophil %**	60.65±8.46 (52)	69.68±13.33 (10)	60.90±14.88 (60)	60.04±11.54 (9)	**0.1502**
**lympho %**	28.89±7.44 (52)	21.78±10.01 (10)	25.32±10.08 (60)	29.8±8.15 (9)	**0.0426**
**mono%**	5.82±1.39 (52)	5.08± 1.99 (10)	5.28±1.93 (60)	5.23±1.53 (9)	**0.2722**
**eosinophil %**	2.54±1.58 (52)	1.46±1.51 (10)	2.24±1.78 (60)	2.93±2.10 (9)	**0.0984**
**basophil %**	0.52±0.20 (51)	0.46±0.32 (10)	0.63±0.55 (60)	0.63±0.38 (9)	**0.8333**
**ESR (mm/h)**	8.80±12.14 (55)	3.80±2.66 (10)	19.85±23.64 (60)	10.25±15.15 (8)	**<0.0001**
**urea (U/L)**	5.62±1.52 (56)	6.09±1.43 (11)	6.18±2.47 (65)	4.82±1.04 (9)	**0.2332**
**creatinine (U/L)**	87.50±15.65 (57)	79.60±20.76 (11)	92.01 ±25.54 (65)	92.99±15.01 (9)	**0.1165**

Summary parameters for age-matched control subjects and the gout patients (Mean±SD (n)). The statistical analysis of laboratory parameters was Kruskal-Wallis test

B		CTRL MV	AU MV	CTRL WT
**ESR**	CTRL WT	**0.0241**	0.6948	-
	AU WT	0.0003	**0.0752**	0.0003
	AU MV	0.2803	-	-
**uric acid**	CTRL WT	**0.244**	<0.0001	-
	AU WT	<0.0001	**0.2476**	<0.0001
	AU MV	0.0012	-	-
**neutrophil**	CTRL WT	**0.0265**	0.8907	-
	AU WT	0.1657	**0.2958**	0.136
	AU MV	0.0947	-	-
**lympho %**	CTRL WT	**0.0186**	0.558	-
	AU WT	0.2562	**0.1634**	0.0572
	AU MV	0.0535	-	-
**eosinophil %**	CTRL WT	**0.0308**	0.6488	-
	AU WT	0.0909	**0.3812**	0.1545
	AU MV	0.1378	-	-
**creatine**	CTRL WT	**0.0299**	0.1406	-
	AU WT	0.0639	**0.4859**	0.6985
	AU MV	0.0562	-	-

Two component statistical analysis of the correlation of the significant laboratory parameters and the presence of the rs55975119 variant in the groups of control individuals and AU patients. The statistical analysis was Mann Whitney U-test. Red is $p < 0.05$. RBC: red blood cells, WBC: white blood cells, ESR: erythrocyte sedimentation rate.

Table S26. Statistical analysis of dual-luciferase assay. The inserted WT or the rs34241868 variant of the *SLC19A3* regions were expressed in HEK293 or MCF cells.

	HEK	MCF
**TK**	0.9953±0.2318	0.4287±0.05
**WT**	0.2699±0.037	0.2679±0.03
**MV**	0.3131±0.018	0.25815±0.036
**p (WT-MV)**	0.0351	0.62

The mean ± SD of ratio of the Renilla/Firefly luciferase activity (n=3). The p values were calculated by T-test with Welch correlation. Red is $p < 0.05$.

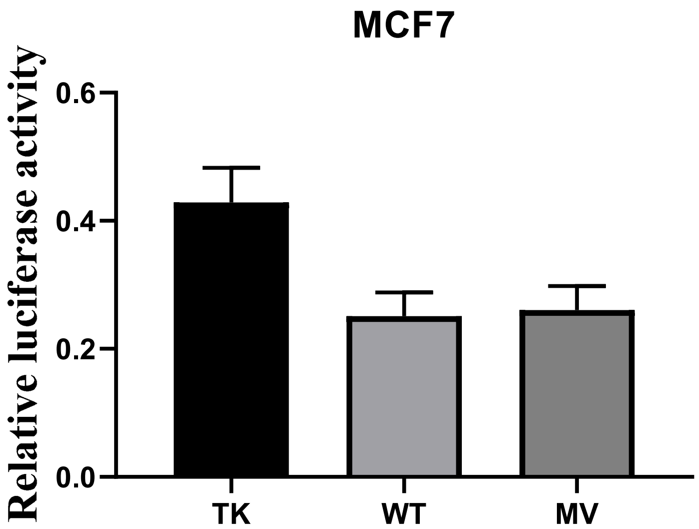

Figure S5. Evaluation of the dual-luciferase assay results. The ratio of the Renilla/normalized Firefly luciferase activity is shown (means +/- SD, n = 3, unpaired two-tailed t-test). The effects of rs3241868 on luciferase activity. The inserted WT or the rs34241868 versions of the *SLC19A3* regions were expressed in MCF7 cells. TK: thymidine kinase promoter-containing control vector, WT: wild type, MV: minor variant.

Table S27. Statistical analysis of effect of treatment. The thiamine depleted HEK cells containing insert of WT or rs34241868 variant were treated by thiamine (B1), fedratinib and metformin.

A	Base	B1	Fedratinib	B1+Fedratinib	Metformin	B1+Metformin
**WT**	0.26361±0.019	0.2477±0.024	0.2599±0.032	0.2667±0.014	0.2715±0.005	0.2364±0.015
**MV**	0.2866±0.018	0.2627±0.041	0.25953±0.034	0.2855±0.027	0.2649±0.013	0.2817±0.020
**p**	0.0539	0.4551	0.9858	0.1761	0.3031	0.0016

Relative luciferase activity mean values and its statistical analysis between wild type and variant group. WT: wild type, MV: minor variant.

B		Thiamine	Fedratinib	Metformin
**WT**	Base	0.2298	0.8127	0.3585
	Thiamine	-	0.644	0.0018
**MV**	Base	0.2287	0.1232	0.0399
	Thiamine	-	0.1764	0.1234

Comparison of the effects of treatments with and without thiamine in the two groups. The mean ± SD of ratio of the Renilla/Firefly luciferase activity (n=3). The p values were calculated by T-test with Welch correlation. Red is $p < 0.05$. WT: wild type, MV: minor variant.

Table S28. Statistical analysis of effect of treatment. The thiamine depleted HEK cells containing insert of WT or rs55975119 variant were treated by thiamine (B1), fedratinib and metformin. TK: thymidine kinase promoter-containing control vector WT: wild type, MV: minor variant.

	HEK	MCF
**TK**	0.9953±0.2318	0.4287±0.05
**WT**	0.2106±0.049	0.2511±0.04
**MV**	0.229±0.044	0.2605±0.037
**p (WT-MV)**	0.5107	0.6700

Relative luciferase activity mean values and its statistical analysis between wild type and variant group. The mean ± SD of ratio of the Renilla/Firefly luciferase activity (n=3). The p values were calculated by T-test with Welch correlation. TK: thymidine kinase promoter-containing control vector, WT: wild type, MV: minor variant.

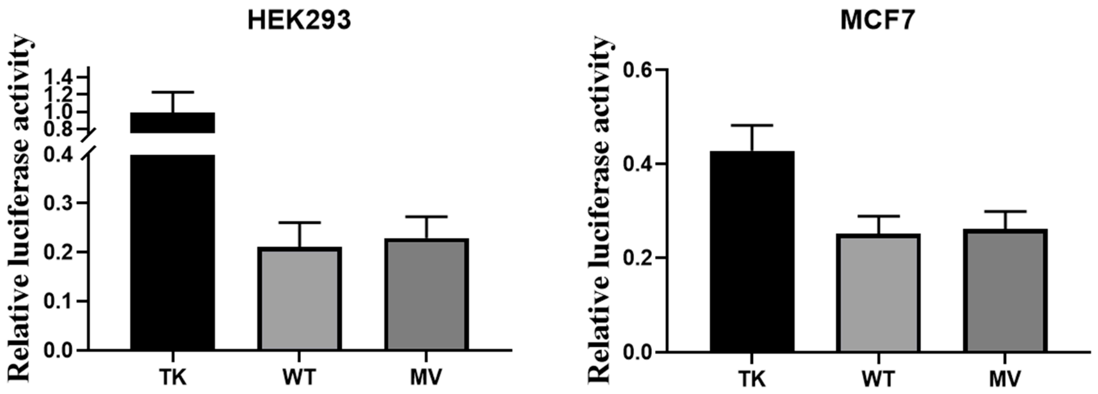

Figure S6. Evaluation of the dual-luciferase assay results. The ratio of the Renilla/normalized Firefly luciferase activity is shown (means +/- SD, n = 3, unpaired two-tailed t-test). The effects of rs55975119 on luciferase activity. The inserted WT or the rs55975119 versions of the *SLC19A3* regions were expressed in HEK293 and MCF7 cells. TK: thymidine kinase promoter-containing control vector, WT: wild type, MV: minor variant.

Table S29. Statistical analysis of effect of treatment. The thiamine depleted HEK cells containing insert of WT or rs55975119 variant were treated by thiamine (B1), fedratinib and metformin.

A	Base	B1	Fedratinib	B1+Fedratinib	Metformin	B1+Metformin
**WT**	0.2531±0.018	0.2615±0.013	0.2548±0.018	0.238±0.0208	0.2535±0.0133	0.2605±0.0195
**MV**	0.2474±0.028	0.2573±0.011	0.2743±0.033	0.2716±0.029	0.2648±0.0173	0.2658±0.0222
**p**	0.6867	0.6148	0.2401	0.04854	0.2344	0.6735

B		Thiamine	Fedratinib	Metformin
**WT**	Base	0.4188	0.8701	0.9652
	Thiamine	-	0.2401	0.4847
**MV**	Base	0.4533	0.1608	0.232
	Thiamine	-	0.8811	0.9364

The mean ± SD of ratio of the Renilla/Firefly luciferase activity (n=3). The p values were calculated by T-test with Welch correlation. Red is $p < 0.05$. WT: wild type, MV: minor variant.

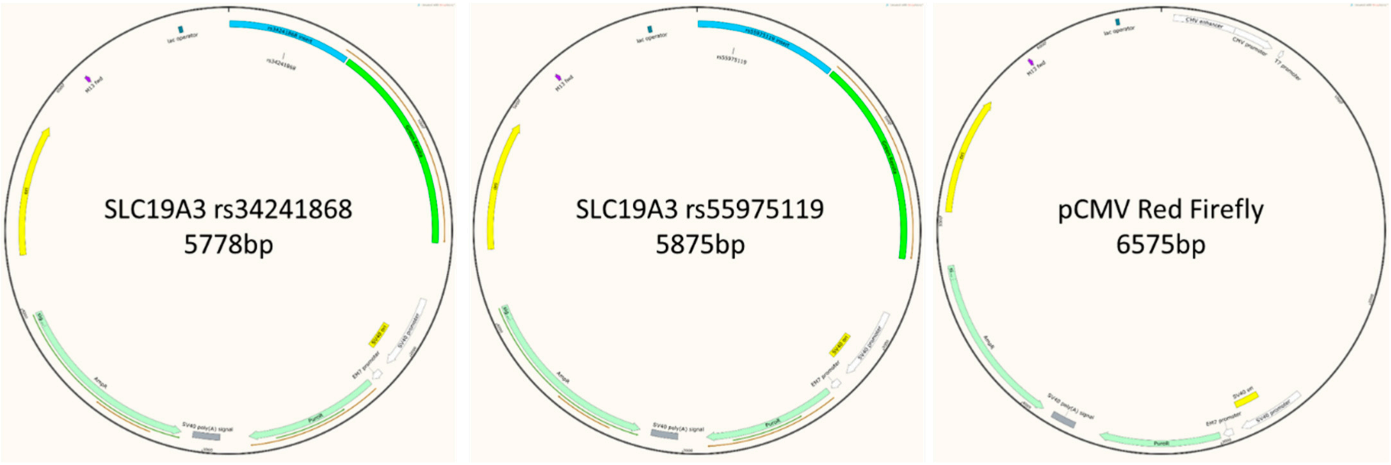

Figure S7. Three types of *SLC19A3* expression vector constructs were used in the dual-luciferase expression assays (generated by SnapGene1.1.3 software.)

Supplementary references on *SLC19A3* SNPs that were not analyzed by us and cannot be classified into haplotypes:

1. Marcé-Grau, A.; Martí-Sánchez, L.; Baide-Mairena, H.; Ortigoza-Escobar, J.D.; Pérez-Dueñas, B. Genetic Defects of Thiamine Transport and Metabolism: A Review of Clinical Phenotypes, Genetics, and Functional Studies. *J. Inherit. Metab. Dis.* **2019**, *42*, 581–597, doi:10.1002/jimd.12125.

2. Porta, M.; Toppila, I.; Sandholm, N.; Hosseini, S.M.; Forsblom, C.; Hietala, K.; Borio, L.; Harjutsalo, V.; Klein, B.E.; Klein, R.; et al. Variation in SLC19A3 and Protection From Microvascular Damage in Type 1 Diabetes. *Diabetes* **2016**, *65*, 1022–1030, doi:10.2337/db15-1247.

3. Bartáková, V.; Pleskačová, A.; Kuricová, K.; Pácal, L.; Dvořáková, V.; Bělobrádková, J.; Tomandlová, M.; Tomandl, J.; Kaňková, K. Dysfunctional Protection against Advanced Glycation Due to Thiamine Metabolism Abnormalities in Gestational Diabetes. *Glycoconj. J.* **2016**, *33*, 591–598, doi:10.1007/s10719-016-9688-9.
